# The Global Burden of Snakebite: A Literature Analysis and Modelling Based on Regional Estimates of Envenoming and Deaths

**DOI:** 10.1371/journal.pmed.0050218

**Published:** 2008-11-04

**Authors:** Anuradhani Kasturiratne, A. Rajitha Wickremasinghe, Nilanthi de Silva, N. Kithsiri Gunawardena, Arunasalam Pathmeswaran, Ranjan Premaratna, Lorenzo Savioli, David G Lalloo, H. Janaka de Silva

**Affiliations:** 1 Faculty of Medicine, University of Kelaniya, Ragama, Sri Lanka; 2 Department of Control of Neglected Tropical Diseases, World Health Organization, Geneva, Switzerland; 3 Liverpool School of Tropical Medicine, Liverpool, United Kingdom; University of Melbourne, Australia

## Abstract

**Background:**

Envenoming resulting from snakebites is an important public health problem in many tropical and subtropical countries. Few attempts have been made to quantify the burden, and recent estimates all suffer from the lack of an objective and reproducible methodology. In an attempt to provide an accurate, up-to-date estimate of the scale of the global problem, we developed a new method to estimate the disease burden due to snakebites.

**Methods and Findings:**

The global estimates were based on regional estimates that were, in turn, derived from data available for countries within a defined region. Three main strategies were used to obtain primary data: electronic searching for publications on snakebite, extraction of relevant country-specific mortality data from databases maintained by United Nations organizations, and identification of grey literature by discussion with key informants. Countries were grouped into 21 distinct geographic regions that are as epidemiologically homogenous as possible, in line with the Global Burden of Disease 2005 study (Global Burden Project of the World Bank). Incidence rates for envenoming were extracted from publications and used to estimate the number of envenomings for individual countries; if no data were available for a particular country, the lowest incidence rate within a neighbouring country was used. Where death registration data were reliable, reported deaths from snakebite were used; in other countries, deaths were estimated on the basis of observed mortality rates and the at-risk population. We estimate that, globally, at least 421,000 envenomings and 20,000 deaths occur each year due to snakebite. These figures may be as high as 1,841,000 envenomings and 94,000 deaths. Based on the fact that envenoming occurs in about one in every four snakebites, between 1.2 million and 5.5 million snakebites could occur annually.

**Conclusions:**

Snakebites cause considerable morbidity and mortality worldwide. The highest burden exists in South Asia, Southeast Asia, and sub-Saharan Africa.

## Abstract 2

Janaka de Silva and colleagues estimate that globally at least 421,000 envenomings and 20,000 deaths occur each year due to snakebite.

## Editors' Summary

**Background.:**

Of the 3,000 or so snake species that exist in the world, about 600 are venomous. Venomous snakes—which exist on every continent except Antarctica—immobilize their prey by injecting modified saliva (venom) that contains toxins into their prey's tissues through their fangs—specialized, hollow teeth. Snakes also use their venoms for self defense and will bite people who threaten, startle or provoke them. Snakebites caused by the families Viperidae (for example, pit vipers) and Elapidae (for example, kraits and cobras) are particularly dangerous to people. The potentially fatal effects of being “envenomed” (having venom injected) by these snakes include widespread bleeding, muscle paralysis, and tissue destruction (necrosis) around the bite site. Bites from these snakes can also cause permanent disability. For example, snakebite victims, who tend to be young and male, may have to have a limb amputated because of necrosis. The best treatment for any snakebite is to get the victim to a hospital as soon as possible where antivenoms (mixtures of antibodies that neutralize venoms) can be given.

**Why Was This Study Done?:**

Although snakebites occur throughout the world, envenoming snakebites are thought to pose a particularly important yet largely neglected threat to public health. This is especially true in rural areas of tropical and subtropical countries where snakebites are common but where there is limited access to health care and to antivenoms. The true magnitude of the public-health threat posed by snakebites in these countries (and elsewhere in the world) is unknown, which makes it hard for public-health officials to optimize the prevention and treatment of snakebites in their respective countries. In this study, therefore, the researchers develop and apply a new method to estimate the global burden of snakebite.

**What Did the Researchers Do and Find?:**

The researchers systematically searched the scientific literature for publications on snakebites and deaths from snakebites and extracted data on snakebite deaths in individual countries from the World Health Organization (WHO) mortality database. They also contacted Ministries of Health, National Poison Centers, and snakebite experts for unpublished information (“grey” literature) on snakebites. Together, these three approaches provided data on the number of snakebite envenomings and deaths for 135 and 162 countries, respectively. The researchers then grouped the 227 countries of the world into 21 geographical regions, each of which contained countries with similar population characteristics, and used the results of studies done in individual countries within each region to estimate the numbers of snakebite envenomings and deaths for each region. Finally, they added up these estimates to obtain an estimate of the global burden of snakebite. Using this method, the researchers estimate that, worldwide, at least 421,000 envenomings and 20,000 deaths from snakebite occur every year; the actual numbers, they suggest, could be as high as 1.8 million envenomings and 94,000 deaths. Their estimates also indicate that the highest burden of snakebite envenomings and death occurs in South and Southeast Asia and in sub-Saharan Africa, and that India is the country with the highest annual number of envenomings (81,000) and deaths (nearly 11,000).

**What Do These Findings Mean?:**

These findings indicate that snakebites cause considerable illness and death around the world. Because of the careful methods used by the researchers, their global estimates of snakebite envenomings and deaths are probably more accurate than previous estimates. However, because the researchers had to make many assumptions in their calculations and because there are so few reliable data on the numbers of snakebites and deaths from the rural tropics, the true regional and global numbers of these events may differ substantially from the estimates presented here. In particular, the regional estimates for eastern sub-Saharan Africa, a region where snakebites are very common and where antivenoms are particularly hard to obtain, are likely to be inaccurate because they are based on a single study. The researchers, therefore, call for more studies on snakebite envenoming and deaths to be done to provide the information needed to deal effectively with this neglected public-health problem.

**Additional Information.:**

Please access these Web sites via the online version of this summary at http://dx.doi.org/10.1371/journal.pmed.0050218.

- This study is further discussed in a *PLoS Medicine* Perspective by Chippaux
- The MedlinePlus Medical Encyclopedia has a page on snakebites (in English and Spanish)
- The UK National Health Service Direct health encyclopedia has detailed information about all aspects of snakebites
- Wikipedia has pages on venomous snakes and on snakebites (note: Wikipedia is a free online encyclopedia that anyone can edit; available in several languages)
- The World Health Organization provides information about antivenoms and about efforts to increase access to antivenoms in developing countries (available in several languages)
- A previous article in *PLoS Medicine* also discusses the neglected problem of snakebite envenoming: Gutiérrez JM, Theakston RDG, Warrell DA (2006) Confronting the Neglected Problem of Snake Bite Envenoming: The Need for a Global Partnership. PLoS Med 3(6): e150

## Introduction

Venomous snakes are found throughout most of the world (including many oceans), except for a few islands, frozen environments, and high altitudes [1]. Envenomings and deaths resulting from snakebites, however, are a particularly important public health problem in the rural tropics. Populations in these regions experience high morbidity and mortality because of poor access to health services, which are often suboptimal, and, in some instances, a scarcity of antivenom, which is the only specific treatment. A large number of victims survive with permanent physical sequelae due to local tissue necrosis and, no doubt, psychological sequelae. Because most snakebite victims are young [2], the economic impact of their disability is considerable. Despite the scale of its effects on populations, snakebite has not received the attention it deserves from national and international health authorities, and may therefore be appropriately categorized as a neglected tropical disease.

Few reliable incidence data are available from the rural tropics where snakebites occur most commonly; reliable data are mostly limited to a few developed countries where bites are rare. Thus, the true global incidence of snakebite envenoming, its impact, and characteristics in different regions remain largely unknown. However, information on the number of bites, envenomings, and deaths and on the frequency of long-term sequelae due to snakebites are essential for assessing the magnitude of the problem, drawing up guidelines for management, planning health care resources (particularly antivenom), and training medical staff to treat snakebites.

Recent estimates, which are fragmentary, variously suggest that worldwide, venomous snakes cause “5.4 million bites, about 2.5 million envenomings and over 125,000 deaths annually” [3], “more than 3 million bites per year resulting in more than 150,000 deaths” [4], or “several million bites and envenomings annually with tens of thousands of deaths” [5]. Since the reviews by Swaroop and Grab in 1954 [6] and Chippaux in 1998 [3], and a global overview of bites and stings from venomous animals by White [4], no comprehensive global assessment has been made of snakebite epidemiology. Swaroop and Grab's review was based mainly on hospital admissions [6], and such data from the rural tropics are fraught with inaccuracies. For example, many snakebite victims in these areas are not hospitalized and seek traditional treatments [7]. Hospital mortality data are well known to underestimate overall mortality due to snakebites [8]. Chippaux [3] and White [4] do not give any details of the methodology used to calculate their estimates. For these reasons, re-estimating the global burden of snakebite using scientifically rigorous, replicable methodologies was necessary.

The objective of this article is to review the currently available literature on snakebite worldwide, and to attempt to make scientifically robust estimates of the current burden. Toward that end, we developed a method to obtain global estimates of envenoming and deaths due to snakebites; these global estimates were based on regional estimates, which were, in turn, derived from data available for countries within a defined region.

## Methods

The methodology consisted of two components, data retrieval and estimation.

### Data Retrieval

The data retrieval process consisted of three main strategies: electronic searching for publications on snakebite; extraction of relevant country specific data required for estimation from databases maintained by the World Health Organization (WHO), United Nations (UN), World Bank (WB), and Food and Agriculture Organization (FAO); and identification of grey literature by discussion with key informants.

#### Publications on snakebite.

**Figure 1:**
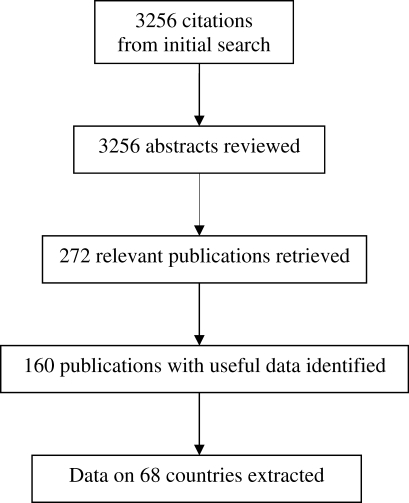
Schematic Diagram of the Steps of the Literature Review

The keywords used for this search were “snakebite and epidemiology,” “snakebite and incidence,” “snakebite and morbidity,” “snakebite and mortality,” “snakebite and envenomation,” “snakebite and envenoming,” and “snakebite and deaths.” The languages of publications included English, French, and Spanish. The process of the published literature review is shown in Figure 1. The original search, carried out at the Liverpool School of Tropical Medicine by a professional data retriever, generated 3,256 citations of publications relating to snakebite. The abstracts of these citations were downloaded and scrutinized by a snakebite expert (DGL) to select all the publications that were likely to be useful for the purpose of the study, considering its objectives. This step resulted in retrieval of 272 full papers. These papers were scrutinized by three independent researchers at the University of Kelaniya to select publications that contained information that would be useful for estimation of incidence and mortality rates of snakebite. Papers reporting extremely high rates from very small studies were not included because they were considered unreliable. This resulted in short-listing 158 original papers and two books.

The data were extracted from the publications, transcribed into data extraction forms, and entered into a database in Microsoft Excel by one researcher. Data from work done earlier than 1985 were excluded from this database. The main variables extracted were: the number of snakebites estimated or reported for a country, nationally, or subnationally; the incidence rate of snakebite; the number of deaths due to snakebite estimated or reported for the entire country; the mortality rate of snakebite; and the percentage of venomous bites out of all snakebites where available. This database was independently cross-checked with the original publications for accuracy and completeness by two other researchers.

When more than one value was available for the same country from a number of sources, only the lowest and the highest values were used. When different rates were quoted in the same publication, the lowest and the highest rates were used. Data for calculation of the number of snakebite envenomings were obtained for 46 countries [9–60] while data for calculation of the number of deaths due to snakebite were obtained for 22 countries [9–11,18, 31,47,49,51,56,58–72] by this process.

#### Databases reviewed.

The WHO Headquarters in Geneva, Switzerland, permitted us access to the WHO mortality database [73] and the WHO population database [74]. The absolute annual number of deaths due to snakebite reported for each country was obtained for the period 1985–2006 from the WHO mortality database [73]. Both versions 9 and 10 of the International Statistical Classification of Diseases and Health Problems (ICD) [75,76] had been used during this period by the reporting countries. The main codes for snakebites under the two versions are ICD-E905.0 (venomous snakes and lizards as the cause of poisoning and toxic reactions) where ICD9 is used, and ICD–T-63.0 (toxic effect of contact with snake venom) in situations where ICD10 is used. We were concerned only with snakebite mortality, which the WHO mortality database tabulates under code X20 (deaths due to venomous snakes and lizards) and not under E and T codes. Thus the data we report for the countries where we obtained information from the WHO mortality database have been tabulated under ICD10 code X20. Annual population estimates by country were obtained from the WHO population database for the 22-y period 1985–2007. For UN countries that are not members of the WHO, the estimates were obtained from the FAO database [77] and UN population database [78]. For each country, the Human Development Index (HDI) for 2005 was obtained from the United Nations Development Programme (UNDP) database [79] and the percentage coverage of vital registration of deaths was obtained from the World Health Statistics Report 2007 [80]. The population living in rural areas for each country reported every 5 y from 1985 to 2005 was extracted from the FAO database [77]. All the variables were double entered and checked.

#### Search for grey literature.

Regional Offices and several country offices of the WHO were contacted to request information on incidence and mortality of snakebite. After the primary review of literature, the countries that did not have reliable published data on snakebite were identified. We contacted Ministries of Health, National Poison Centres, researchers, and experts on snakebite in these countries for information on the incidence of, and mortality due to, snakebite in their countries.

A second search on the internet using the Google search engine was conducted for all countries still without data. The keywords used for this search were the “(country name) and snakebite.” This search included sources in English, Japanese, and Russian.

### Estimation

Two hundred twenty-seven countries were grouped into 21 distinct geographical regions in this study, according to the classification used for the Global Burden of Disease (GBD) 2005 study (Global Burden Project of the World Bank) [81]. This latest iteration of the GBD study is led by the new Institute for Health Metrics and Evaluation at the University of Washington, with key collaborating institutions including Harvard University, WHO, Johns Hopkins University, and the University of Queensland, and is funded by the Bill and Melinda Gates Foundation. The 21 regions have been defined with two objectives: first, to define regions that are as epidemiologically homogeneous as possible, so that information from detailed studies in one country can plausibly be extrapolated to other countries in the region; and second, related to the first, to create burden estimates that are useful to individual countries in planning for health sector activities. The regions were chosen using mortality estimates from the WHO and UN, in addition to what is known about country-specific epidemiological conditions. The 21-region classification was adopted because this study also has the same objectives, albeit in relation to a single disease condition (snakebite envenoming), and for the sake of consistency with the GBD methodology. The 227 countries were listed and categorized into two groups, i.e., countries in which snakebite occurs and countries in which snakebite does not occur. This categorization was done by content experts, based on the availability of evidence to support the occurrence of recent snakebite in each country.

#### Assumptions.

A country was considered free of snakebites (and associated mortality) if no literature (published or unpublished) indicated the occurrence of snakebite since 1985. A country was considered to have no mortality due to snakebites, even though snakebites have been reported, if no mortality statistics have been reported to the WHO mortality database from 1990 to date, provided that the country has a HDI 0.750 or higher and vital registration coverage of deaths 75% or higher. The most recent incidence or mortality rate that had been calculated after 1985 was assumed to be the current rate for a country. If the HDI was 0.750 or higher and coverage of vital registration of deaths was 75% or higher, the average number of deaths reported over the last 5 y to the WHO mortality database in the respective category of the ICD code was assumed to be the total number of deaths due to snakebite for the country.

The assumptions made on the representativeness of data were based on the national coverage of the data. It was assumed that the different types of data (data originating from different sources) were representative in the following order of priority. (1) Published community-based national data; (2) published hospital-based national data; (3) unpublished national data (community/hospital based); (4) published community-based subnational data; (5) unpublished community based subnational data; and (6) published subnational hospital data where the population served was known.

High and low estimates of the number of envenomings and deaths due to snakebite for each GBD region were derived from the highest and lowest estimated number of envenomings and deaths for countries within a region. Regional estimates were summed up to arrive at global estimates.

#### Calculation and estimation of the incidence rates and number of snakebite envenomings (Figure 2).

Four types of data were available to be used for calculation of the incidence rate of snakebite envenoming (per 100,000 population) for each country. These data were prioritised as follows. (1) Published data or estimates of the absolute number of annual snakebites in a country and the number of persons seeking treatment for snakebite each year, reported by National Ministries of Health, National Poison Centres, or the entire hospital system in a country;(2) unpublished data or estimates of the absolute number of annual snakebites in a country and the number of persons seeking treatment for snakebite each year, reported by National Ministries of Health, National Poison Centres, or the entire hospital system in a country; (3) number of snakebites based on the incidence reported in published community-based studies at the subnational level; and (4) number of snakebites based on the incidence reported in published hospital-based studies at the subnational level.

**Figure 2:**
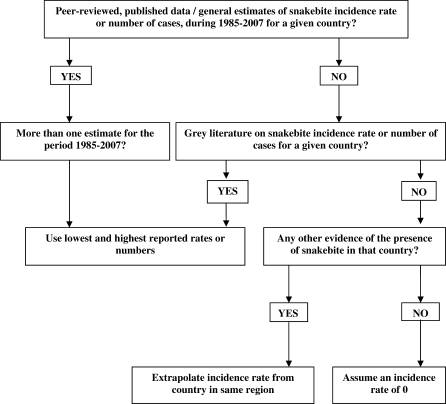
Algorithm for Calculation of Morbidity Due to Snakebite

Incidence rates calculated from nationwide community-based studies were not available in the literature. Where the absolute annual number of envenomings for a given country was available, the incidence rate was calculated using the country population for the reporting year as the denominator. For subnational data, the incidence rate was calculated using the population surveyed or the population served by the hospitals, as the denominator.

The following steps were then followed. For countries where snakebites do not occur, a zero incidence was used. For countries having a single national incidence rate, the rate was applied to the total population to estimate the number of snakebites for the country. For countries having a single subnational incidence rate, the rate was applied to the subnational study population, and the number obtained was considered the number of snakebites for the entire country.

Two incidence rates (high and low) were calculated for countries for which more than one rate was available. The lower rate was applied to the total population of the country for the low estimate. The high estimate was obtained by summing the estimates obtained after applying the higher incidence rate to the rural population and the lower rate to the urban population of that country.

For countries where snakebite is known to occur, but which did not have data, two estimates of the number of snakebites occurring within the country were calculated as follows. The low estimate was calculated by applying the lowest incidence rate reported by another country within the same GBD 2005 region to the given country's population. The high estimate was calculated by summing the estimates obtained after applying the highest incidence rate reported within the same GBD 2005 region to the given country's rural population and the lowest incidence rate reported within the same region to the given country's urban population.

#### Calculation and estimation of mortality rates and number of deaths due to snakebites (Figure 3).

For countries where snakebites do not occur the number of deaths due to snakebites was estimated as zero. For countries that had a HDI of 0.750 or more in 2005 [79], and the rate of vital registration of deaths was 75% or more for the last year of reporting [80], the average of the number of deaths reported annually for the latest 5 y was estimated to be the number of deaths for 2007. For countries that had a HDI below 0.750 or a vital registration rate below 75%, the absolute number of deaths due to snakebite was calculated based on literature and other sources of information. The cut-off points for HDI and percentage vital registration of deaths were set at 0.750 and 75%, respectively, being the upper limit of the interquartile range

**Figure 3:**
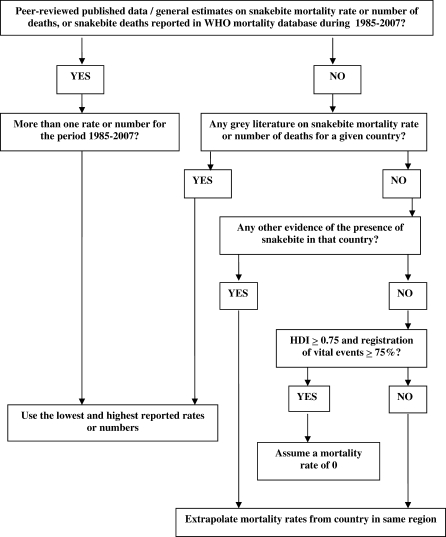
Algorithm for Calculation of Mortality Due to Snakebite

The following steps were used in the estimation of deaths due to snakebites. Mortality rates (per 100,000 population) were calculated using the country populations of the reporting year as the denominator. If only one mortality rate was available, it was considered the lower mortality rate. If more than one mortality rate was available for the same country, the lowest and the highest reported rates were considered the low and high mortality rates. For subnational data, the mortality rate was calculated using the population surveyed, or the population served by the hospitals, as the denominator. Within each GBD 2005 region the lowest and the highest mortality rates reported were applied to countries without data.

The following method was used for estimation of the number of deaths due to snakebite for each country. For countries where snakebites do not occur, zero mortality was used. For countries having national mortality rates, the rate was applied to the total population of that country to arrive at the estimate of the number of deaths due to snakebites. For countries having subnational mortality rates, the higher mortality rate was applied to the rural population of that country and the lower mortality rate was applied to the urban population of that country, and the two estimates were summed to arrive at the high estimate for that country.

#### Estimation of total snakebite burden.

The majority (70%) of the published papers on snakebite described only snakebite envenomings, while a very few (*n* = 14) described both total snakebite and snakebite resulting in envenoming. A considerable number (*n* = 32) did not make a distinction between bites resulting in envenoming and bites not resulting in envenoming. Although our prime estimate was snakebite envenomings, we also estimated the total number of snakebites, using figures for the percentage of bites with envenoming out of total bites reported in a few publications. The calculated number of bites without envenoming was added to the number of bites with envenoming estimated originally.

## Results

The review of published literature generated 3,256 citations on snakebite. Data on incidence and/or mortality of snakebite were available in 160 publications.

### Estimation of Incidence Rate and the Number of Snakebite Envenomings

**Figure 4:**
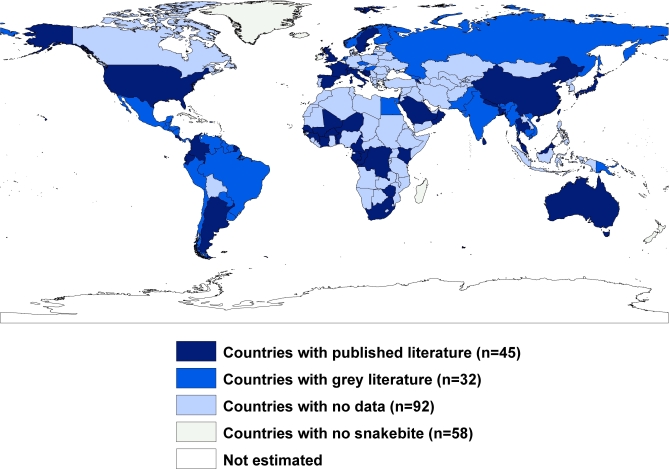
Countries with Data on Snakebite Envenoming

**Figure 5:**
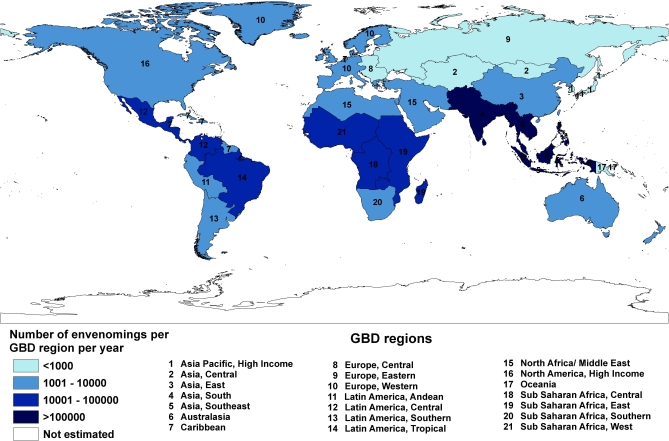
Regional Estimates of Envenomings Due to Snakebite (Low Estimate)

Of the 227 countries, 58 were identified as countries where venomous snakebites do not occur. Data useful for calculation of the incidence of snakebite in 62 countries were found in 40 publications [9–60]. The review of grey literature resulted in two Web sources [82,83] and 13 communications that generated data for 15 more countries. Data thus obtained for 77 countries were used for the estimation of the number of snakebite envenomings in 92 countries without data (Figure 4; Table S1). The estimated number of snakebite envenomings by region is shown in Figure 5 and Table 1. In our most conservative estimate, the highest number of envenomings were estimated for South Asia (121,000) followed by Southeast Asia (111,000), and East Sub-Saharan Africa (43,000). The lowest numbers were estimated for Central Europe and Central Asia. We estimate that, globally, at least 421,000 envenomings occur annually; this figure may be as high as 1,841,000. According to our most conservative country estimates, which were used to calculate the regional estimates, India had the most envenomings at 81,000 per year. Sri Lanka (33,000), Viet Nam (30,000), Brazil (30,000), Mexico (28,000), and Nepal (20,000) were the other countries that had a high estimated number of envenomings annually.

**Table 1:**
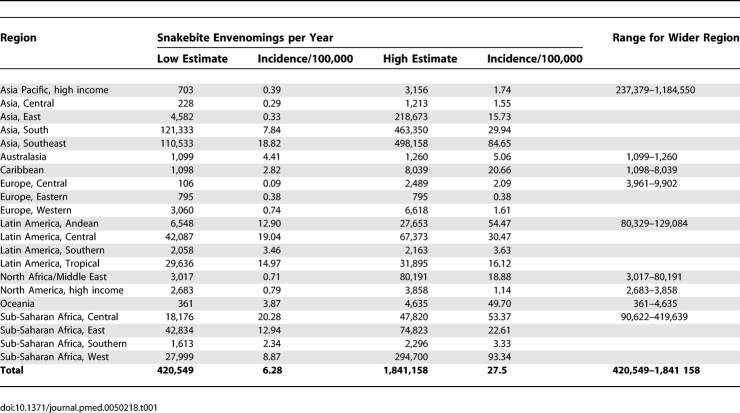
Global Estimates of the Snakebite Envenomings in 2007 by Region

### Estimation of Mortality Rate and Number of Deaths Due to Snakebites

**Figure 6:**
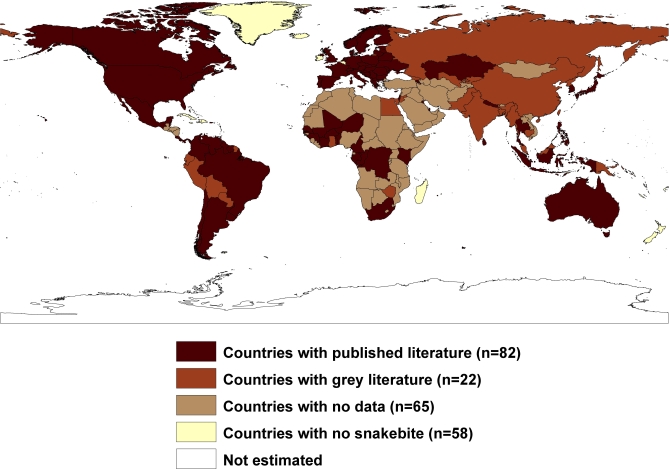
Countries with Data on Snakebite Mortality

**Figure 7:**
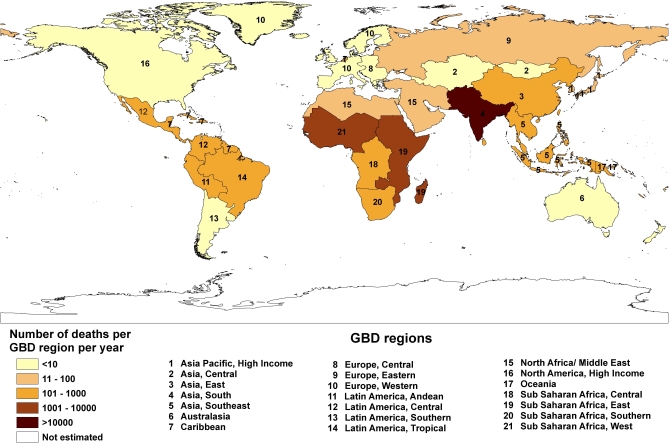
Regional Estimates of Deaths Due to Snakebite (Low Estimate)

Seventy-four countries had a HDI 0.750 or higher [79] and a death registration rate 75% or higher [80]. Of these, 15 countries were classified as not having snakebites. Among the rest, 27 countries had no deaths due to snakebite according to the WHO mortality database [73]. The other 32 countries reported deaths due to snakebite, which were used for calculation of the country-specific mortality rates. Among countries with a lower HDI or a lower rate of registration, mortality rates were obtained from published literature for 23 countries [9–11,18,31,47,49,51, 56,58–72]. Grey literature provided mortality rates for 22 more countries. When the other 43 countries without snakebites were excluded, no snakebite mortality data existed for 65 countries. The number of deaths due to snakebite in these 65 countries was estimated based on the 104 countries for which mortality data were available (Figure 6; Table S2). The estimated number of snakebite deaths by region are shown in Table 2 and Figure 7. In our most conservative estimate the highest number of deaths due to snakebite was estimated in South Asia (14,000) followed by West sub-Saharan Africa (1,500) and East sub-Saharan Africa (1,400). The lowest numbers of deaths were estimated for Australasia, Southern Latin America, and Western Europe. We estimate that globally, at least 20,000 deaths occur from snakebite annually; this figure may be as high as 94,000. According to our most conservative country estimates that were used to calculate the regional estimates, India had the highest number of deaths due to snakebite in the world with nearly 11,000 deaths annually. Bangladesh and Pakistan had over 1,000 deaths per year.

**Table 2:**
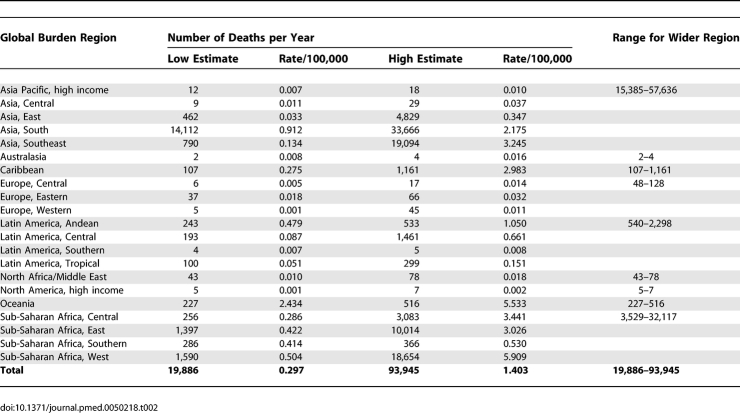
Global Estimates of Deaths Due to Snakebites in 2007 by Region

### Estimation of the Total Number of Snakebites (Envenomed and Nonenvenomed Bites)

Even bites by nonvenomous snakes or bites by a venomous snake that do not cause envenoming may pose a burden on health systems, because in some regions victims access the health care system and require assessment. We tried to estimate the total number of snakebites (envenomed and nonenvenomed) by looking at the literature in different continents to enable us to extrapolate from the number of envenomings. In Asia, various studies suggest that envenomed bites constitute between 12% and 50% of the total number of bites [17,84,85]. The most complete data suggest that envenomed bites constitute 18% and 30% of the total in India and Pakistan, respectively (Ian Simpson, personal communication). Data are limited for North and Latin America: in Brazil, 56% of the snakebites were caused by nonvenomous snakes [86], and American Association of Poison Control Centers data suggest that the total number of snakebites is about three times that of venomous bites [37]. African data are equally variable; 19% of snakebite victims in Kenya were bitten by potentially venomous snakes [7], and two studies in West Africa suggested that envenoming made up 45% and 87% of total bites [60,87].

These data indicate that the relationship of the total number of snakebites to envenoming is highly variable and may be influenced by a number of factors. To give an indication of the total number of bites, we have assumed that the total number of snakebites would be two to three times the number of envenomings. This estimate combined with our estimate that the number of envenomings ranges from 421,000 to as high as 1,841,000 annually, we estimate 1,200,000 to 5,500,000 snakebites may occur globally per year.

## Discussion

We estimate that at least 421,000 envenomings and 20,000 deaths occur worldwide from snakebite annually. These figures may be as high as 1,841,000 envenomings and 94,000 deaths. On the basis of the estimation that the total number of snakebites is two to three times the number of envenomings**,** we estimate that 1,200,000–5,500,000 snakebites may occur globally. The vast majority of the estimated burden of snakebite is in South and Southeast Asia, sub-Saharan Africa, and Central and South America, as identified in previous estimates of the global burden. Despite accounting for nearly one-fourth of the global snakebite incidence, mortality due to snakebite is relatively lower in Central and South America when compared to other high incidence regions. Mortality may be lower because of better snakebite management systems, including the development of locally effective antivenoms, in many Latin American countries. The lower estimates of snakebite incidence in sub-Saharan Africa are probably a reflection of under-reporting from many parts of this region; we found it particularly difficult to find reliable data for this region, especially for East Africa. India, with its population of over a billion people, accounted for the highest estimated number of bites and deaths for a single country.

The most often quoted currently available estimates of the global burden of snakebite [3,4] are subject to the major limitation that the methodology of estimation is not given and so cannot be reproduced. The formalization of methods for the assessment of disease burden provides a framework for standardized methodology [81]. In an attempt to provide a more contemporary and accurate picture of the global problem, we developed and applied a method to obtain an estimate of the disease burden due to snakebite. Our global estimates were based on regional estimates that were, in turn, derived from data available for countries within a defined region.

The true global incidence of snakebite, envenomings, and its associated mortality are difficult to estimate. The overwhelming majority of bites occur in rural areas of resource-poor countries. Reporting and record-keeping in such situations are generally poor. Snakebite varies seasonally and geographically within countries; i.e., high incidences are reported during agricultural activity [88]. Many estimates from these countries are based on hospital returns or incomplete central databases, and are bound to be underestimates, because many victims do not seek hospital treatment and prefer traditional remedies [5]. Some may die at home, with their deaths unrecorded [8]. Studies from rural Nigeria and Kenya have reported that only 8.5% and 27% of snakebite victims, respectively, sought hospital treatment [7,89]. This situation may be common to many middle- and low-income countries where health-seeking behaviour, health beliefs, and access to health care are not optimal. Thus, most of the available data on snakebite should be regarded as underestimates.

Conversely, many of the few published community surveys of snakebite have been performed in areas where the problem is endemic and perhaps a major public health problem, and incidence and mortality figures then extrapolated to represent the entire country or region; this would lead to an overestimation of the burden. To circumvent this problem we applied the higher rates identified for a particular country only to the rural population of that country if the rates were reported at subnational level. This would have resulted in considerable underestimation, because some of the published subnational studies have reportedly been conducted in regions where snakebite is not considered a high priority public health problem [59,60,90,91]. It is for these reasons, and because the paucity of data prevented more precise estimations, that we decided to present our estimates as a range by calculating both high and low estimates of snakebites and related mortality.

The most important issue we faced was the paucity of good-quality published data, particularly from nationwide community-based studies. Despite this deficiency, we have given priority to national estimates derived from the national health systems and related services over rates available from subnational studies to try to avoid overestimation of national incidence or mortality rates. Data generated from hospital-based studies were used in a few instances where no other data were available, provided that the catchment population was known.

We considered only data reported after 1985 to make the estimates as current as possible. More than 70% of the data that were utilised in arriving at the estimates were, in fact, reported after 1995. Most regions lacked either population-based studies or surveillance systems that might measure snakebite incidence at the population level. In some cases, many countries in a region lacked data. For example, data were available for only one country in eastern sub-Saharan Africa, where snakebite is known to be an important public health problem. Population-based studies of incidence and mortality are urgently needed to describe the epidemiology of snakebite in these areas. In some countries that encompass large geographical areas and have large populations such as India, China, Indonesia, and Russia, country estimates had to be calculated on the basis of single or few regional incidence or mortality figures. This also means that relatively small changes in the incidence rates could lead to considerable differences in the estimation of the total burden in terms of the number of envenomings and deaths. The proportion of the population that is rural, and therefore exposed to the risk of snakebites, can also considerably affect estimates for these countries.

On the rare occasions where multiple studies were reported for the same country, we selected the most conservative incidence and mortality rates for our low estimates. We followed this principle when extrapolating data for countries within a region. That is, where no data were available for a country in which bites were likely to occur, we selected the most conservative rate available from a country within the region as the rate for the country without data [92]. This was done to avoid bias toward overestimation of the incidence and mortality in the region where the study was conducted. This is especially relevant for countries such as China, India, and Indonesia which have very large populations. When calculating our high estimate we used only the higher figures of incidence to calculate burden for the rural population of a given country (where snakebites are commoner) and considered the lowest incidence figures when calculating the burden in the urban population in that country. Thus, even our high estimates could be considered conservative.

Most previous estimates of snakebite morbidity and mortality appear to have been derived from studies that were done in such high incidence areas within regions or countries, and extrapolated to whole countries and regions. Our approach minimised this effect, although in some regions, such as the Caribbean, lack of data still meant that we were forced to use very high rates in our calculations of the high estimate. These methodological differences may have played a substantial role in accounting for the differences between the previous estimates and our low estimate (Table 3). The case for this is further strengthened by the fact that those estimates are closer to our high estimate. However, this is difficult to assess given that a clear methodology is not available for any of the previous estimates.

**Table 3:**
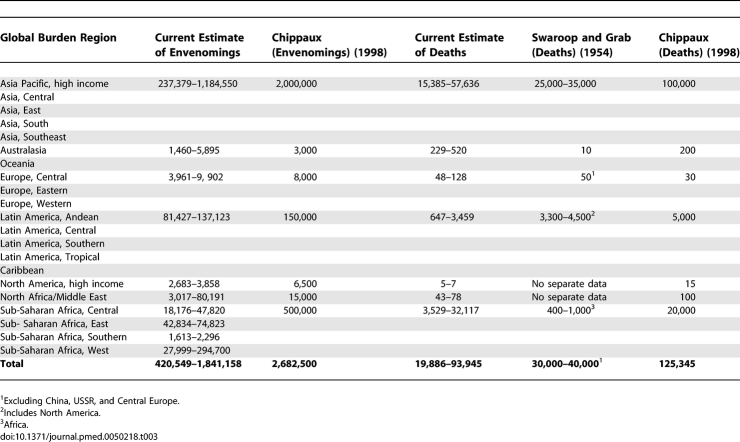
Comparison between the Current and Previous Estimates of Snakebite Envenomings and Deaths

Another plausible reason is that we used very recent incidence and mortality data from India and Pakistan that are considerably lower than previous estimates. Our estimate of about 81,000 envenomings and nearly 11,000 deaths due to snakebite in India is much lower than the 200,000 bites and up to 50,000 deaths quoted in previous estimates [63]. Given the population of over one billion in India, this had a substantial effect on our global estimates. The figures we report from India are based on health insurance schemes operated by many state governments such as the “Kisan Jeevan Kalyan Yojana” (Ian Simpson, personal communication). These schemes compensate the farming community for a variety of accidents, including snakebite. The level of compensation is substantial, ranging from US$250 to US$1,250. We feel that these figures may therefore be a more reliable estimate than hospital records, because the victim's family has an incentive to report the deaths due to snakebites, reducing the assumed impact of unreported deaths. It is possible that unexplained deaths are attributed to snakebite, but victims are examined and mortality certified by a doctor as being due to snakebite.

Estimation of the total number of snakebites (both with and without envenoming) is difficult because of the scarcity of literature that differentiates the two and variation in the distribution of venomous snakes in the regions; few community-based studies address this. The true burden of snakebites may not be reflected in hospital data, because a considerable proportion of people with asymptomatic bites may not seek treatment at hospitals; in some settings, snakebite victims may preferentially attend traditional healers. It was also not possible to ascertain whether “all bites,” especially in community surveys, included bites of nonvenomous snakes and dry bites of venomous snakes. These non-envenoming bites would arguably not contribute much to the burden of disease, although the opportunity cost of the bite may affect the victims and the households adversely. We estimated the total number of snakebites using data on the proportion of snakebites with envenoming from studies in different parts of the world. The proportion envenomed varied considerably, most probably because of both the effects of different snake species and variation in methodology. This heterogeneity means that we have only a crude estimate of the total number of snakebites. Our main focus was to estimate snakebite envenoming, as it is envenoming that causes most of the burden due to snakebite: requirement for antivenom, hospital and intensive care unit care, and surgery; complications; permanent sequelae; and even death.

The WHO mortality database was the main source of information used for estimation of the mortality due to snakebite, providing nearly 70% of the data used. The reliability of the data reported to this database by countries was assessed on the basis of two criteria: the HDI and the coverage of vital registration of deaths. Only code X20 of ICD10 was considered snakebite to minimise inclusion of deaths caused by other venomous animals. Code X20 does not differentiate between deaths due to snakes and lizards. However, lizard bites, unlike venomous snakebites, are not known to be fatal; in fact, in the last 50 y only a few cases of envenoming and no deaths have been reported as due to lizard bites. Thus, from an epidemiological point of view lizard bites are legible and have no public health implications.

In any estimation, assumptions have to be made and the robustness and validity of the estimate depends on how well the assumptions are met. The assumptions we made included considering a country as free of snakebites (and associated mortality) if there was no literature (published or unpublished) indicating the occurrence of snakebite since 1985, and assuming that the mortality data reported to the WHO mortality database to be accurate and representative of the national data for countries with a high HDI and a high vital registration coverage of deaths. We did not make any adjustments to arrive at the final estimate of deaths for countries with less than 100% vital registration coverage to prevent an apparent unrealistic precision to the estimate. The most recent incidence or mortality rate reported after 1985 was assumed to be the current rate for a country.

We estimated the numbers of snakebites and deaths only. No reliable data were available on the long-term physical and psychological consequences of surviving snakebite, but as most snakebite victims are in the economically productive age group, the economic impact of disability is likely to be high. Although the socioeconomic burden of snakebite cannot be stressed strongly enough, in this study we did not attempt to quantify this burden. Global health resource allocation is often based on DALYs (disability-adjusted life-years), and other socioeconomic markers rather than on the number of patients and deaths, despite the limitations of each of these measures of burden. Future assessments of the burden of snakebite will draw greater attention to the problem, which in turn will help win resources to tackle this neglected issue.

In conclusion, data from several new sources and the development and application of a scientifically robust method that can be replicated has enabled us to generate a revised estimate of the global disease burden due to snakebite, although the inadequacy of available data and the consequent need to rely upon extrapolation mean that this estimate is still far from perfect. The burden is considerable, especially in South and Southeast Asia, sub-Saharan Africa, and Central and South America. Given the high burden, the paucity of reliable snakebite data, particularly in some of these areas, is both surprising and worrying. The fact that snakebite varies geographically and seasonally, that it is mainly a rural tropical phenomenon where reporting and record keeping is poor, and that health-seeking behaviour is diverse with traditional treatments being sometimes preferred to Western medicine, all contribute to the difficulties faced when studying its epidemiology.

To address this problem, population-based studies of incidence and mortality in countries that appear to have the highest case load and mortality rates are urgently required to clarify the situation. The quality of reporting and recordkeeping on morbidity and mortality due to snakebite in health facilities should be optimised. Data sources should include traditional medical practitioners and rural health workers, because high morbidity and mortality due to snakebite can be encountered in geographically isolated communities [9]. Accurate data on the epidemiology of snakebite, globally, will facilitate prioritisation of scarce health care resources for prevention and treatment of this neglected health problem.

## Supporting Information

Table S1 Estimation of the Total Number of Snakebite Envenomings by Country (427 KB DOC)

Table S2 Estimation of the Total Number of Deaths Due to Snakebite by Country (478 KB DOC)

## Abbreviations

FAO: Food and Agriculture Organization
GBD: Global Burden of Disease
HDI: Human Development Index
UN: United Nations
UNDP: United Nations Development Programme
WB: World Bank
WHO: World Health Organization
