# Supplementary material for: The Global Burden of Snakebite: A Literature Analysis and Modelling Based on Regional Estimates of Envenoming and Deaths
Source: PLoS Med. 2008 Nov 4;5(11):e218. doi: 10.1371/journal.pmed.0050218 (PMC2577696; doi:10.1371/journal.pmed.0050218)
Supplement: Supplementary file 1 [file pmed.0050218.st001.doc]

**Table S1. Estimation of the total number of snake bite envenomings by country**

| **Global Burden Region** | **Country** | **Incidence per**  **100 000 population (Lower)** | **Source** | **Incidence per**  **100 000 population (Higher)** | **Source** | **Current population** | **Number of cases (Low)** | **Number of cases (High)** |
| --- | --- | --- | --- | --- | --- | --- | --- | --- |
| Asia Pacific, High income | Brunei Darussalam | 0.3980 | estimate | 4.9960 | estimate | 390056 | 2 | 6 |
| Asia Pacific, High income | Japan | 0.3980 | Warrell, 1995 | 4.9960 | website+ * | 127966709 | 509 | 2529 |
| Asia Pacific, High income | Republic of Korea | 0.3980 | estimate | 4.9960 | estimate | 48223853 | 192 | 621 |
| Asia Pacific, High income | Singapore | 0.0000 | N | 0.0000 | N | 4436281 | 0 | 0 |
| Asia, Central | Armenia | 2.4590 | website ** | 2.4590 | web | 3002271 | 74 | 74 |
| Asia, Central | Azerbaijan | 0.2050 | estimate | 2.4590 | estimate | 8467167 | 17 | 115 |
| Asia, Central | Georgia | 0.2050 | GL | 2.4590 | estimate | 4395420 | 9 | 63 |
| Asia, Central | Kazakhstan | 0.2050 | estimate | 2.4590 | estimate | 15421861 | 32 | 186 |
| Asia, Central | Kyrgyzstan | 0.2050 | estimate | 2.4590 | estimate | 5316543 | 11 | 91 |
| Asia, Central | Mongolia | 0.2050 | estimate | 2.4590 | estimate | 2628840 | 5 | 32 |
| Asia, Central | Tajikistan | 0.2050 | estimate | 2.4590 | estimate | 6735996 | 14 | 125 |
| Asia, Central | Turkmenistan | 0.2050 | estimate | 2.4590 | estimate | 4965278 | 10 | 73 |
| Asia, Central | Uzbekistan | 0.2050 | estimate | 2.4590 | estimate | 27372260 | 56 | 453 |
| Asia, East | China | 0.0850 | Warrell, 1995§ | 26.6000 | Peinan et al. 1989+ | 1336317116 | 1136 | 212118 |
| Asia, East | DPR Korea | 0.0850 | estimate | 26.6000 | estimate | 23790245 | 20 | 2359 |
| Asia, East | Hong Kong | 6.0500 | Cockram et al. 1990 | 26.6000 | estimate | 7057000 | 427 | 427 |
| Asia, East | Taiwan | 13.1200 | Chen et al. 2000 | 26.6000 | estimate | 22858872 | 2999 | 3769 |
| Asia, South | Afghanistan | 2.5300 | estimate | 604.0000 | estimate | 27145275 | 687 | 128738 |
| Asia, South | Bangladesh | 2.5300 | Sarker et al. 1999+ | 6.1300 | Sarker et al. 1999+ | 158664959 | 4014 | 8280 |
| Asia, South | Bhutan | 373.3000 | GL | 604.000 | estimate | 658479 | 2458 | 2977 |
| Asia, South | India | 6.9630 | GL | 17.1000 | GL | 1169015509 | 81399 | 163051 |
| Asia, South | Nepal | 70.9000 | Devkota et al. 2001 | 604.0000 | Sharma et al 2004+ | 28195994 | 19991 | 142727 |
| Asia, South | Pakistan | 7.8000 | GL | 12.2000 | GL | 163902405 | 12784 | 17578 |
| Asia, Southeast | Cambodia | 5.5000 | GL | 173.5000 | estimate | 14443679 | 794 | 21482 |
| **Global Burden Region** | **Country** | **Incidence per**  **100 000 population (Lower)** | **Source** | **Incidence per**  **100 000 population (Higher)** | **Source** | **Current population** | **Number of cases (Low)** | **Number of cases (High)** |
| Asia, Southeast | Indonesia | 5.5000 | estimate | 173.5000 | estimate | 231626978 | 12739 | 214883 |
| Asia, Southeast | Lao, Peoples Republic of | 5.5000 | estimate | 173.5000 | estimate | 5859393 | 322 | 8383 |
| Asia, Southeast | Malaysia | 44.4540 | Warrell, 1995 | 173.5000 | estimate | 26571879 | 11812 | 23637 |
| Asia, Southeast | Maldives | 0.0000 | N | 0.0000 | N | 305556 | 0 | 0 |
| Asia, Southeast | Mauritius | 0.0000 | N | 0.0000 | N | 1261641 | 0 | 0 |
| Asia, Southeast | Mayotte | 0.0000 | N | 0.0000 | N | 0 | 0 | 0 |
| Asia, Southeast | Myanmar | 17.6800 | GL | 20.6700 | GL | 48798212 | 8832 | 14633 |
| Asia, Southeast | Philippines | 5.5000 | estimate | 173.5000 | estimate | 87960117 | 4838 | 58989 |
| Asia, Southeast | Seychelles | 0.0000 | N | 0.0000 | N | 86606 | 0 | 0 |
| Asia, Southeast | Sri Lanka | 173.5000 | Ministry of Health 2007 | 173.5000 | Ministry of Health, 2007 | 18963553 | 32902 | 32902 |
| Asia, Southeast | Thailand | 13.2500 | GL | 14.7000 | GL | 63883662 | 8465 | 9101 |
| Asia, Southeast | Timor Leste | 5.5000 | estimate | 173.5000 | estimate | 1154775 | 64 | 1501 |
| Asia, Southeast | Viet Nam | 34.3000 | GL | 173.5000 | estimate | 87375196 | 29970 | 117583 |
| Australasia | Australia | 5.3000 | Currie 2000 | 16.0000 | Currie 2000 | 20743179 | 1099 | 1260 |
| Australasia | New Zealand | 0.0000 | N | 0.0000 | N | 4178525 | 0 | 0 |
| Caribbean | Anguilla | 0.0000 | N | 0.0000 | N | 12000 | 0 | 0 |
| Caribbean | Antigua and Barbuda | 0.0000 | N | 0.0000 | N | 85109 | 0 | 0 |
| Caribbean | Aruba | 48.5000 | GL | 600.0000 | estimate | 103000 | 50 | 364 |
| Caribbean | Bahamas | 0.0000 | N | 0.0000 | N | 331278 | 0 | 0 |
| Caribbean | Barbados | 0.0000 | N | 0.0000 | N | 293891 | 0 | 0 |
| Caribbean | Belize | 17.8000 | GL | 600.0000 | estimate | 287699 | 51 | 884 |
| Caribbean | Bermuda | 0.0000 | N | 0.0000 | N | 83000 | 0 | 0 |
| Caribbean | British Virgin Islands | 0.0000 | N | 0.0000 | N | 22000 | 0 | 0 |
| Caribbean | Cayman Islands | 0.0000 | N | 0.0000 | N | 43000 | 0 | 0 |
| Caribbean | Cuba | 0.0000 | N | 0.0000 | N | 11267883 | 0 | 0 |
| Caribbean | Dominica | 0.0000 | N | 0.0000 | N | 67390 | 0 | 0 |
| Caribbean | Dominican Republic | 0.0000 | N | 0.0000 | N | 9759664 | 0 | 0 |
| **Global Burden Region** | **Country** | **Incidence per**  **100 000 population (Lower)** | **Source** | **Incidence per**  **100 000 population (Higher)** | **Source** | **Current population** | **Number of cases (Low)** | **Number of cases (High)** |
| Caribbean | French Guiana | 45.0000 | Chippaux & Theakston 1987 | 600.0000 | Chippaux & Theakston 1987 | 187000 | 84 | 334 |
| Caribbean | Grenada | 0.0000 | N | 0.0000 | N | 105672 | 0 | 0 |
| Caribbean | Guadeloupe | 0.0000 | N | 0.0000 | N | 446000 | 0 | 0 |
| Caribbean | Guyana | 71.5000 | GL | 600.0000 | estimate | 737906 | 528 | 3017 |
| Caribbean | Haiti | 0.0000 | N | 0.0000 | N | 9597849 | 0 | 0 |
| Caribbean | Jamaica | 0.0000 | N | 0.0000 | N | 2713779 | 0 | 0 |
| Caribbean | Martinique | 5.4000 | Thomas et al. 1996 | 12.5900 | PC | 397000 | 20 | 21 |
| Caribbean | Montserrat | 0.0000 | N | 0.0000 | N | 4000 | 0 | 0 |
| Caribbean | Netherland Antilles | 0.0000 | N | 0.0000 | N | 224000 | 0 | 0 |
| Caribbean | Saint Kitts and Nevus | 0.0000 | N | 0.0000 | N | 50417 | 0 | 0 |
| Caribbean | Saint Lucia | 2.5000 | GL | 600.0000 | estimate | 164924 | 4 | 640 |
| Caribbean | Saint Vincent and Grenadine | 0.0000 | N | 0.0000 | N | 120402 | 0 | 0 |
| Caribbean | Suriname | 71.5000 | GL | 600.0000 | estimate | 457964 | 327 | 868 |
| Caribbean | Trinidad and Tobago | 2.5000 | GL | 600.0000 | estimate | 1333272 | 33 | 1911 |
| Caribbean | Turks and Caicos Island | 0.0000 | N | 0.0000 | N | 22000 | 0 | 0 |
| Europe, Central | Albania | 0.0300 | estimate | 5.2000 | estimate | 3190012 | 1 | 94 |
| Europe, Central | Bosnia & Herzegovina | 0.3000 | Bubalo et al. 2004§ | 2.7000 | Bubalo et al. 2004+ | 3934816 | 12 | 67 |
| Europe, Central | Bulgaria | 0.0300 | estimate | 5.2000 | estimate | 7638831 | 2 | 119 |
| Europe, Central | Croatia | 0.5700 | Luksic et al. 2006§ | 5.2000 | Luksic et al. 2006+ | 4555398 | 26 | 108 |
| Europe, Central | Czech Republic | 0.3000 | GL | 5.2000 | estimate | 10186330 | 31 | 158 |
| Europe, Central | Hungary | 0.03000 | GL | 5.2000 | estimate | 10029683 | 3 | 174 |
| Europe, Central | Montenegro | 0.0300 | estimate | 5.2000 | estimate | 597983 | 0 | 0 |
| Europe, Central | Poland | 0.0300 | estimate | 5.2000 | estimate | 38081971 | 11 | 765 |
| Europe, Central | Romania | 0.0300 | estimate | 5.2000 | estimate | 21437887 | 6 | 523 |
| Europe, Central | Serbia | 0.0300 | estimate | 5.2000 | estimate | 9858424 | 3 | 262 |
| Europe, Central | Slovakia | 0.0300 | estimate | 5.2000 | estimate | 5390035 | 9 | 124 |
| **Global Burden Region** | **Country** | **Incidence per**  **100 000 population (Lower)** | **Source** | **Incidence per**  **100 000 population (Higher)** | **Source** | **Current population** | **Number of cases (Low)** | **Number of cases (High)** |
| Europe, Central | Slovenia | 0.0300 | estimate | 5.2000 | estimate | 2001506 | 1 | 51 |
| Europe, Central | TFYR Macedonia | 0.0300 | estimate | 5.2000 | estimate | 2038464 | 1 | 44 |
| Europe, Eastern | Belarus | 0.3800 | estimate | 0.3800 | estimate | 9688795 | 37 | 37 |
| Europe, Eastern | Estonia | 0.3800 | estimate | 0.3800 | estimate | 1335333 | 5 | 5 |
| Europe, Eastern | Latvia | 0.3800 | estimate | 0.3800 | estimate | 2277040 | 9 | 9 |
| Europe, Eastern | Lithuania | 0.3800 | estimate | 0.3800 | estimate | 3389937 | 13 | 13 |
| Europe, Eastern | Republic of Moldova | 0.3800 | estimate | 0.3800 | estimate | 3793604 | 14 | 14 |
| Europe, Eastern | Russian Federation | 0.3800 | GL§ | 0.3800 | GL§ | 142498532 | 541 | 541 |
| Europe, Eastern | Ukraine | 0.3800 | estimate | 0.380 | estimate | 46205382 | 176 | 176 |
| Europe, Western | Andorra | 0.0900 | estimate | 5.2060 | estimate | 74601 | 0 | 0 |
| Europe, Western | Austria | 0.0900 | estimate | 5.2060 | estimate | 8360746 | 8 | 151 |
| Europe, Western | Belgium | 0.0000 | N | 0.0000 | N | 10457343 | 0 | 0 |
| Europe, Western | Channel Islands | 0.0000 | N | 0.0000 | N | 149000 | 0 | 0 |
| Europe, Western | Cyprus | 0.0900 | estimate | 5.2060 | estimate | 854671 | 1 | 14 |
| Europe, Western | Denmark | 0.0900 | estimate | 5.2060 | estimate | 5442098 | 5 | 45 |
| Europe, Western | Faro Islands | 0.0000 | N | 0.0000 | N | 47000 | 0 | 0 |
| Europe, Western | Finland | 3.8000 | GL | 5.2060 | estimate | 5276892 | 201 | 229 |
| Europe, Western | France | 2.5000 | Persson, 1995 | 4.8700 | Persson, 1995 | 61647375 | 1541 | 1880 |
| Europe, Western | Germany | 0.0900 | estimate | 5.2060 | estimate | 82599471 | 74 | 561 |
| Europe, Western | Gibraltar | 0.0000 | N | 0.0000 | N | 27000 | 0 | 0 |
| Europe, Western | Greece | 0.0900 | estimate | 5.2060 | estimate | 11146918 | 10 | 228 |
| Europe, Western | Greenland | 0.0000 | N | 0.0000 | N | 57000 | 0 | 0 |
| Europe, Western | Holy See | 0.0000 | N | 0.0000 | N | 1000 | 0 | 0 |
| Europe, Western | Iceland | 0.0000 | N | 0.0000 | N | 301006 | 0 | 0 |
| Europe, Western | Ireland | 0.0000 | N | 0.0000 | N | 4300902 | 0 | 0 |
| Europe, Western | Isle of Man | 0.0000 | N | 0.0000 | N | 78000 | 0 | 0 |
| Europe, Western | Israel | 1.4400 | Abraham et al. 2001 | 4.8300 | Abraham et al. 2001 | 6927677 | 100 | 119 |
| Europe, Western | Italy | 0.7900 | Pozio 1988 | 5.2060 | estimate | 58876834 | 465 | 1290 |
| Europe, Western | Liechtenstein | 0.0000 | N | 0.0000 | N | 34000 | 0 | 0 |
| Europe, Western | Luxembourg | 0.0000 | N | 0.0000 | N | 466560 | 0 | 0 |
| Europe, Western | Malta | 0.0000 | N | 0.0000 | N | 406582 | 0 | 0 |
| **Global Burden Region** | **Country** | **Incidence per**  **100 000 population (Lower)** | **Source** | **Incidence per**  **100 000 population (Higher)** | **Source** | **Current population** | **Number of cases (Low)** | **Number of cases (High)** |
| Europe, Western | Monaco | 0.0000 | N | 0.0000 | N | 32711 | 0 | 0 |
| Europe, Western | Netherlands | 0.0900 | GL | 5.2060 | estimate | 16418824 | 15 | 293 |
| Europe, Western | Norway | 0.8500 | GL | 5.2060 | estimate | 4698097 | 40 | 79 |
| Europe, Western | Portugal | 0.0900 | estimate | 5.2060 | estimate | 10623031 | 10 | 240 |
| Europe, Western | San Marino | 0.0900 | estimate | 5.2060 | estimate | 6000 | 0 | 0 |
| Europe, Western | San Pierre et Miquelon | 0.0000 | N | 0.0000 | N | 30926 | 0 | 0 |
| Europe, Western | Spain | 0.2522 | GL | 5.2060 | Persson, 1995 | 44279180 | 112 | 597 |
| Europe, Western | Sweden | 2.6000 | Karlson-Stiber et al. 2006 | 5.2060 | estimate | 9118955 | 237 | 276 |
| Europe, Western | Switzerland | 0.5750 | GL | 2.7000 | Petite 2005+ | 7483973 | 43 | 93 |
| Europe, Western | United Kingdom | 0.3290 | GL | 5.2060 | estimate | 60768946 | 200 | 522 |
| Latin America, Andean | Bolivia | 8.9900 | estimate | 146.4000 | estimate | 9524568 | 856 | 5500 |
| Latin America, Andean | Ecuador | 8.9900 | GL | 146.4000 | Praba-Egge et al.2003+ | 13341197 | 1199 | 8189 |
| Latin America, Andean | Peru | 16.1000 | GL | 146.4000 | estimate | 27902760 | 4492 | 13963 |
| Latin America, Central | Colombia | 5.0700 | Otero et al. 2002 | 8.4300 | Otero et al. 2002 | 46155958 | 2340 | 2696 |
| Latin America, Central | Costa-Rica | 12.8000 | GL | 20.0000 | Rojas et al. 1997+ | 4467625 | 572 | 695 |
| Latin America, Central | El Salvador | 0.7390 | GL | 76.0400 | estimate | 6857328 | 51 | 2122 |
| Latin America, Central | Guatemala | 3.8400 | GL | 76.0400 | estimate | 13353911 | 513 | 5715 |
| Latin America, Central | Honduras | 7.1700 | GL | 76.0400 | estimate | 7106001 | 510 | 3294 |
| Latin America, Central | Mexico | 26.3350 | Frayre-Torres et al. 2006 | 76.0400 | estimate | 106534880 | 28 056 | 41009 |
| Latin America, Central | Nicaragua | 9.0400 | GL | 14.5000 | GL | 5603190 | 507 | 641 |
| Latin America, Central | Panama | 76.0400 | GL | 76.0400 | estimate | 3343374 | 2542 | 2542 |
| Latin America, Central | Venezuela | 25.3000 | GL | 76.0400 | estimate | 27656832 | 6997 | 8659 |
| Latin America, Southern | Argentina | 4.9500 | Cardoso, 1995 | 4.9500 | estimate | 39531115 | 1957 | 1957 |
| Latin America, Southern | Chile | 0.0050 | GL | 4.9500 | estimate | 16634760 | 1 | 102 |
| Latin America, Southern | Falkland Islands | 0.0000 | N | 0.0000 | N | 3000 | 0 | 0 |
| Latin America, Southern | Uruguay | 3.0000 | GL | 4.9500 | estimate | 3339700 | 100 | 105 |
| **Global Burden Region** | **Country** | **Incidence per**  **100 000 population (Lower)** | **Source** | **Incidence per**  **100 000 population (Higher)** | **Source** | **Current population** | **Number of cases (Low)** | **Number of cases (High)** |
| Latin America, Tropical | Brazil | 15.4000 | GL | 21.2800 | GL | 191790929 | 29536 | 31274 |
| Latin America, Tropical | Paraguay | 1.6300 | GL | 21.2800 | estimate | 6127077 | 100 | 621 |
| North Africa/ Middle East | Algeria | 0.2950 | estimate | 47.2390 | estimate | 33857915 | 100 | 6470 |
| North Africa/ Middle East | Bahrain | 0.2950 | estimate | 47.2390 | estimate | 752648 | 2 | 38 |
| North Africa/ Middle East | Egypt | 0.2950 | GL | 47.2390 | estimate | 75497913 | 223 | 21254 |
| North Africa/ Middle East | Iran | 0.2950 | estimate | 47.2390 | estimate | 71208384 | 210 | 11079 |
| North Africa/ Middle East | Iraq | 0.2950 | estimate | 47.2390 | estimate | 28993374 | 86 | 4366 |
| North Africa/ Middle East | Jordan | 0.2950 | estimate | 47.2390 | estimate | 5924245 | 17 | 613 |
| North Africa/ Middle East | Kuwait | 0.2950 | estimate | 47.2390 | estimate | 2851144 | 8 | 56 |
| North Africa/ Middle East | Lebanon | 0.2950 | estimate | 47.2390 | estimate | 4099115 | 12 | 228 |
| North Africa/ Middle East | Libyan Arab Jamahiriya | 0.2950 | estimate | 47.2390 | estimate | 6160483 | 18 | 388 |
| North Africa/ Middle East | Morocco | 0.2950 | estimate | 47.2390 | estimate | 31224137 | 92 | 6349 |
| North Africa/ Middle East | Occupied Palestinian Territory | 0.2950 | estimate | 47.2390 | estimate | 3762000 | 11 | 513 |
| North Africa/ Middle East | Oman | 13.9020 | GL | 47.2390 | Hanssens et al. 2001 | 2595133 | 361 | 583 |
| North Africa/ Middle East | Qatar | 0.2950 | estimate | 47.2390 | estimate | 840635 | 2 | 26 |
| North Africa/ Middle East | Saudi Arabia | 6.0000 | Mahaba 2000+ | 18.0000 | Mahaba 2000+ | 24734533 | 1484 | 1855 |
| North Africa/ Middle East | Syrian Arab Republic | 0.2950 | estimate | 47.2390 | estimate | 19928516 | 59 | 4651 |
| North Africa/ Middle East | Tunisia | 0.2950 | estimate | 47.2390 | estimate | 10327285 | 30 | 1747 |
| North Africa/ Middle East | Turkey | 0.2950 | estimate | 47.2390 | estimate | 74876695 | 221 | 11777 |
| North Africa/ Middle East | United Arab Emirates | 0.2950 | estimate | 47.2390 | estimate | 4380439 | 13 | 239 |
| North Africa/ Middle East | Western Sahara | 0.2950 | estimate | 47.2390 | estimate | 324000 | 1 | 10 |
| North Africa/ Middle East | Yemen | 0.2950 | estimate | 47.2390 | estimate | 22389169 | 66 | 7949 |
| North America, High income | Canada | 0.7920 | estimate | 2.6000 | estimate | 32876047 | 260 | 372 |
| North America, High income | USA | 0.7920 | Gold et al. 2002 | 2.6000 | Gold et al. 2002 | 305826246 | 2422 | 3487 |
| Oceania | American Samoa | 0.0000 | N | 0.0000 | N | 65000 | 0 | 0 |
| Oceania | Cook Islands | 0.0000 | N | 0.0000 | N | 13325 | 0 | 0 |
| Oceania | Fiji | 0.0000 | N | 0.0000 | N | 838698 | 0 | 0 |
| **Global Burden Region** | **Country** | **Incidence per**  **100 000 population (Lower)** | **Source** | **Incidence per**  **100 000 population (Higher)** | **Source** | **Current population** | **Number of cases (Low)** | **Number of cases (High)** |
| Oceania | French Polynesia | 0.0000 | N | 0.0000 | N | 252000 | 0 | 0 |
| Oceania | Guam | 0.0000 | N | 0.0000 | N | 168000 | 0 | 0 |
| Oceania | Kiribati | 0.0000 | N | 0.0000 | N | 95067 | 0 | 0 |
| Oceania | Marshall Islands | 0.0000 | N | 0.0000 | N | 59286 | 0 | 0 |
| Oceania | Micronesia (Federated States of) | 4.9500 | estimate | 74.2500 | estimate | 111117 | 6 | 60 |
| Oceania | Nauru | 0.0000 | N | 0.0000 | N | 10152 | 0 | 0 |
| Oceania | New Caledonia | 0.0000 | N | 0.0000 | N | 237000 | 0 | 0 |
| Oceania | Niue | 4.9500 | estimate | 74.2500 | estimate | 1565 | 0 | 1 |
| Oceania | Northern Mariana Islands | 0.0000 | N | 0.0000 | N | 86000 | 0 | 0 |
| Oceania | Palau | 4.9500 | estimate | 74.2500 | estimate | 20314 | 1 | 6 |
| Oceania | Papua NG | 4.9500 | GL§ | 74.2500 | GL§ | 6331010 | 313 | 4051 |
| Oceania | Pitcairn Islands | 0.0000 | N | 0.0000 | N | 0 | 0 | 0 |
| Oceania | Samoa | 0.0000 | N | 0.0000 | N | 187023 | 0 | 0 |
| Oceania | Solomon Islands | 4.9500 | estimate | 74.2500 | estimate | 495662 | 25 | 328 |
| Oceania | Tokelau | 0.0000 | N | 0.0000 | N | 2000 | 0 | 0 |
| Oceania | Tonga | 4.9500 | estimate | 74.2500 | estimate | 100336 | 5 | 54 |
| Oceania | Tuvalu | 0.0000 | N | 0.0000 | N | 10530 | 0 | 0 |
| Oceania | Vanuatu | 4.9500 | estimate | 74.2500 | estimate | 226180 | 11 | 135 |
| Oceania | Wallis and Futuna Islands | 0.0000 | N | 0.0000 | N | 15000 | 0 | 0 |
| Sub Saharan Africa, Central | Angola | 7.4130 | estimate | 77.2790 | estimate | 17024084 | 1262 | 8004 |
| Sub Saharan Africa, Central | Central African Republic | 7.4130 | estimate | 77.2790 | estimate | 4342735 | 322 | 1935 |
| Sub Saharan Africa, Central | Congo | 11.5000 | Carme et al. 1986+ | 20.0000 | Carme et al. 1986+ | 3768086 | 435 | 593 |
| Sub Saharan Africa, Central | DR Congo | 25.5780 | Bokata 2005+ | 77.2790 | Bokata 2005+ | 62635723 | 16021 | 36814 |
| Sub Saharan Africa, Central | Equatorial Guinea | 7.4130 | estimate | 77.2790 | estimate | 507499 | 38 | 229 |
| Sub Saharan Africa, Central | Gabon | 7.4130 | Tchoua et al. 2002 | 77.2790 | estimate | 1330574 | 99 | 246 |
| Sub Saharan Africa, East | Burundi | 13.8000 | estimate | 28.7070 | estimate | 8508232 | 1174 | 2230 |
| Sub Saharan Africa, East | Comoros | 0.0000 | N | 0.0000 | N | 839187 | 0 | 0 |
| Sub Saharan Africa, East | Djibouti | 13.8000 | estimate | 28.7070 | estimate | 833021 | 115 | 132 |
| **Global Burden Region** | **Country** | **Incidence per**  **100 000 population (Lower)** | **Source** | **Incidence per**  **100 000 population (Higher)** | **Source** | **Current population** | **Number of cases (Low)** | **Number of cases (High)** |
| Sub Saharan Africa, East | Eritrea | 13.8000 | estimate | 28.7070 | estimate | 4850763 | 669 | 1233 |
| Sub Saharan Africa, East | Ethiopia | 13.8000 | estimate | 28.7070 | estimate | 83099190 | 11468 | 21223 |
| Sub Saharan Africa, East | Kenya | 13.8000 | Coombs et al. 1997+ | 28.7070 | Chippaux 2005+ | 37537716 | 5180 | 8194 |
| Sub Saharan Africa, East | Madagascar | 0.0000 | N | 0.0000 | N | 19683358 | 0 | 0 |
| Sub Saharan Africa, East | Malawi | 13.8000 | estimate | 28.7070 | estimate | 13925070 | 1922 | 3556 |
| Sub Saharan Africa, East | Mozambique | 13.8000 | estimate | 28.7070 | estimate | 21396916 | 2953 | 4832 |
| Sub Saharan Africa, East | Rwanda | 13.8000 | estimate | 28.7070 | estimate | 9724577 | 1342 | 2399 |
| Sub Saharan Africa, East | Somalia | 13.8000 | estimate | 28.7070 | estimate | 8698534 | 1200 | 2289 |
| Sub Saharan Africa, East | Sudan | 13.8000 | estimate | 28.7070 | estimate | 38560488 | 5321 | 8554 |
| Sub Saharan Africa, East | Uganda | 13.8000 | estimate | 28.7070 | estimate | 30883805 | 4262 | 8109 |
| Sub Saharan Africa, East | United Republic of Tanzania | 13.8000 | estimate | 28.7070 | estimate | 40453513 | 5583 | 9341 |
| Sub Saharan Africa, East | Zambia | 13.8000 | estimate | 28.7070 | estimate | 11921999 | 1645 | 2731 |
| Sub Saharan Africa, South | Botswana | 1.1240 | estimate | 5.3300 | estimate | 1881504 | 21 | 58 |
| Sub Saharan Africa, South | Lesotho | 1.1240 | estimate | 5.3300 | estimate | 2007833 | 23 | 85 |
| Sub Saharan Africa, South | Namibia | 1.1240 | estimate | 5.3300 | estimate | 2074146 | 23 | 82 |
| Sub Saharan Africa, South | South Africa | 2.8480 | Blaylock 2004§ | 5.3300 | Blaylock 2004§ | 48576763 | 1383 | 1864 |
| Sub Saharan Africa, South | Swaziland | 1.1240 | estimate | 5.3300 | estimate | 1141427 | 13 | 48 |
| Sub Saharan Africa, South | Zimbabwe | 1.1240 | Kasilo & Nhachi§ 1993 | 1.2380 | Nhachi & Kasilo 1994§ | 13349434 | 150 | 160 |
| Sub Saharan Africa, West | Benin | 65.4590 | Fayomi et al. 2002 | 200.000 | Chippaux 2002+ | 9032787 | 5913 | 11392 |
| Sub Saharan Africa, West | Burkina Faso | 7.5000 | Chippaux 2005+ | 67.6390 | Chippaux 2005 | 14784291 | 1109 | 8279 |
| Sub Saharan Africa, West | Cameroon | 12.1560 | Chippaux et al. 2002§ | 60.7830 | Chippaux et al. 2002§ | 18549176 | 2255 | 6206 |
| Sub Saharan Africa, West | Cape Verde | 0.0000 | N | 0.0000 | N | 530437 | 0 | 0 |
| Sub Saharan Africa, West | Chad | 0.8100 | estimate | 200.0000 | estimate | 10780571 | 87 | 14414 |
| Sub Saharan Africa, West | Cote d’Ivoire | 33.1240 | Chippaux 2002 | 95.1860 | Chippaux 2002 | 19261821 | 6380 | 12366 |
| Sub Saharan Africa, West | Gambia | 0.8100 | estimate | 200.0000 | estimate | 1708681 | 14 | 2344 |
| Sub Saharan Africa, West | Ghana | 0.8100 | estimate | 200.0000 | estimate | 23478394 | 190 | 24515 |
| **Global Burden Region** | **Country** | **Incidence per**  **100 000 population (Lower)** | **Source** | **Incidence per**  **100 000 population (Higher)** | **Source** | **Current population** | **Number of cases (Low)** | **Number of cases (High)** |
| Sub Saharan Africa, West | Guinea-Bissau | 0.8100 | estimate | 200.0000 | estimate | 1695043 | 14 | 2170 |
| Sub Saharan Africa, West | Guinea | 13.6090 | Balde et al. 2002 | 100.0000 | Chippaux 2005 | 9370113 | 1275 | 6295 |
| Sub Saharan Africa, West | Liberia | 0.8100 | estimate | 200.0000 | estimate | 3750261 | 30 | 4104 |
| Sub Saharan Africa, West | Mali | 0.8100 | Chippaux 2005 | 200.0000 | estimate | 12336799 | 100 | 19516 |
| Sub Saharan Africa, West | Mauritania | 0.8100 | estimate | 200.0000 | estimate | 3123813 | 25 | 2325 |
| Sub Saharan Africa, West | Niger | 9.4000 | Chippaux & Kambewasso 2002+ | 200.0000 | estimate | 14225521 | 1337 | 21527 |
| Sub Saharan Africa, West | Nigeria | 0.8100 | estimate | 200.0000 | estimate | 148092542 | 1200 | 141710 |
| Sub Saharan Africa, West | Saint Helena | 0.8100 | estimate | 200.0000 | estimate | 5000 | 0 | 6 |
| Sub Saharan Africa, West | Sao Tome and Principe | 0.8100 | estimate | 200.0000 | estimate | 157638 | 1 | 217 |
| Sub Saharan Africa, West | Senegal | 10.0000 | Chippaux & Diallo 2002+ | 23.0000 | Chippaux & Diallo 2002+ | 12378532 | 1238 | 1946 |
| Sub Saharan Africa, West | Sierra Leone | 0.8100 | estimate | 200.0000 | estimate | 5865872 | 48 | 6724 |
| Sub Saharan Africa, West | Togo | 103.0000 | Chippaux 2005+ | 157.0000 | Chippaux 2005+ | 6585147 | 6783 | 8644 |

GL- Grey literature	N- Countries without snakebite	§ Number reported in a region extrapolated for the entire country	+ Regional rates used directly

* [http://www3.pref.okinawa.jp/site/contents/attach/12559/6-6.pdf#search](http://www3.pref.okinawa.jp/site/contents/attach/12559/6-6.pdf#search)= ** <http://www.panorama.am/en/health/2007/08/24/ashtarak>
