# Supplementary material for: The Global Burden of Snakebite: A Literature Analysis and Modelling Based on Regional Estimates of Envenoming and Deaths
Source: PLoS Med. 2008 Nov 4;5(11):e218. doi: 10.1371/journal.pmed.0050218 (PMC2577696; doi:10.1371/journal.pmed.0050218)
Supplement: Supplementary file 2 [file pmed.0050218.st002.doc]

**Table S2. Estimation of the total number of deaths due to snake bite by country**

| **Global Burden Region** | **Country** | **Mortality rate per 100,000 population (Lower)** | **Source** | **Mortality rate per 100,000 population (Higher)** | **Source** | **Current Population** | **Number of deaths (Low)** | **Number of deaths (High)** |
| --- | --- | --- | --- | --- | --- | --- | --- | --- |
| Asia Pacific, High income | Brunei Darussalam | 0.0000 | WHO* | 0.0000 | WHO* | 390056 | 0.0 | 0.0 |
| Asia Pacific, High income | Japan | 0.0058 | WHO* | 0.0104 | estimate | 127966709 | 7.4 | 13.3 |
| Asia Pacific, High income | Republic of Korea | 0.0104 | WHO* | 0.0104 | WHO* | 48223853 | 5.0 | 5.0 |
| Asia Pacific, High income | Singapore | 0.0000 | N | 0.0000 | N | 4436281 | 0.0 | 0.0 |
| Asia, Central | Armenia | 0.0000 | WHO* | 0.0000 | WHO* | 3002271 | 0.0 | 0.0 |
| Asia, Central | Azerbaijan | 0.0110 | estimate | 0.0517 | estimate | 8467167 | 0.9 | 4.4 |
| Asia, Central | Georgia | 0.0000 | GL | 0.0000 | GL | 4395420 | 0.0 | 0.0 |
| Asia, Central | Kazakhstan | 0.0000 | WHO * | 0.0000 | WHO* | 15421861 | 0.0 | 0.0 |
| Asia, Central | Kyrgyzstan | 0.0517 | GL | 0.0517 | GL | 5316543 | 2.8 | 2.8 |
| Asia, Central | Mongolia | 0.0110 | estimate | 0.0517 | estimate | 2628840 | 0.3 | 1.4 |
| Asia, Central | Tajikistan | 0.0110 | estimate | 0.0517 | estimate | 6735996 | 0.7 | 3.5 |
| Asia, Central | Turkmenistan | 0.0110 | estimate | 0.0517 | estimate | 4965278 | 0.5 | 2.6 |
| Asia, Central | Uzbekistan | 0.0110 | GL | 0.0517 | estimate | 27372260 | 3.0 | 14.2 |
| Asia, East | China | 0.0337 | GL | 0.4900 | Peinan et al. 1989 | 1336317116 | 450.0 | 4081.0 |
| Asia, East | Democratic People’s Republic of Korea | 0.0000 | GL | 0.0000 | GL | 23790245 | 0.0 | 0.0 |
| Asia, East | Hong Kong | 0.0750 | Cockram et al. 1990 | 2.5000 | estimate | 7057000 | 5.3 | 176.4 |
| Asia, East | Taiwan | 0.0315 | Chen et al. 2000 | 2.5000 | Cheng & Winkel 2001 | 22858872 | 7.2 | 571.5 |
| Asia, South | Afghanistan | 0.7093 | estimate | 5.0421 | estimate | 27145275 | 192.5 | 1368.7 |
| Asia, South | Bangladesh | 1.0588 | GL | 5.0421 | GL | 158664959 | 1680.0 | 8000.0 |
| Asia, South | Bhutan | 0.7093 | estimate | 5.0421 | estimate | 658479 | 4.7 | 33.2 |
| Asia, South | India | 0.9268 | GL | 1.2831 | Warrell, 1993 | 1169015509 | 10835.0 | 15000.0 |
| Asia, South | Nepal | 0.7093 | WHO 1987 | 3.5466 | WHO 1987 | 28195994 | 200.0 | 1000.0 |
| Asia, South | Pakistan | 0.7321 | GL | 5.0421 | estimate | 163902405 | 1200.0 | 8264.1 |
| Asia, Southeast | Cambodia | 0.5539 | GL | 5.0000 | estimate | 14443679 | 80.0 | 722.2 |
| Asia, Southeast | Indonesia | 0.0086 | WHO, 2005 | 5.0000 | estimate | 231626978 | 20.0 | 11581.3 |
| Asia, Southeast | Lao, Peoples Republic of | 0.0078 | estimate | 5.0000 | estimate | 5859393 | 0.5 | 293.0 |
| **Global Burden Region** | **Country** | **Mortality rate per 100,000 population (Lower)** | **Source** | **Mortality rate per 100,000 population (Higher)** | **Source** | **Current Population** | **Number of deaths (Low)** | **Number of deaths (High)** |
| Asia, Southeast | Malaysia | 0.0301 | GL | 0.3000 | Meier & White 1995 | 26571879 | 8.0 | 79.7 |
| Asia, Southeast | Maldives | 0.0000 | N | 0.0000 | N | 305556 | 0.0 | 0.0 |
| Asia, Southeast | Mauritius | 0.0000 | N | 0.0000 | N | 1261641 | 0.0 | 0.0 |
| Asia, Southeast | Mayotte | 0.0000 | N | 0.0000 | N | 186452 | 0.0 | 0.0 |
| Asia, Southeast | Myanmar | 0.6900 | GL | 1.3300 | GL | 48798212 | 336.7 | 649.0 |
| Asia, Southeast | Philippines | 0.2274 | WHO, 2005 | 0.3411 | WHO, 2005 | 87960117 | 200.0 | 300.0 |
| Asia, Southeast | Seychelles | 0.0000 | N | 0.0000 | N | 86606 | 0.0 | 0.0 |
| Asia, Southeast | Sri Lanka | 0.7000 | Ministry of Health 2007 | 5.0000 | Warrell 1993 | 18963553 | 132.7 | 948.2 |
| Asia, Southeast | Thailand | 0.0078 | WHO, 2005 | 0.1471 | WHO* | 63883662 | 5.0 | 94.0 |
| Asia, Southeast | Timor Leste | 0.0078 | estimate | 5.0000 | estimate | 1154775 | 0.1 | 57.7 |
| Asia, Southeast | Viet Nam | 0.0078 | estimate | 5.0000 | estimate | 87375196 | 6.8 | 4368.8 |
| Australasia | Australia | 0.0084 | WHO* | 0.0200 | Cheng & Winkel 2001 | 20743179 | 1.8 | 4.1 |
| Australasia | New Zealand | 0.0000 | N | 0.0000 | N | 4178525 | 0.0 | 0.0 |
| Caribbean | Anguilla | 0.0000 | N | 0.0000 | N | 12000 | 0.0 | 0.0 |
| Caribbean | Antigua and Barbuda | 0.0000 | N | 0.0000 | N | 85109 | 0.0 | 0.0 |
| Caribbean | Aruba | 0.1390 | estimate | 53.4800 | estimate | 103000 | 0.1 | 55.1 |
| Caribbean | Bahamas | 0.0000 | N | 0.0000 | N | 331278 | 0.0 | 0.0 |
| Caribbean | Barbados | 0.0000 | N | 0.0000 | N | 293891 | 0.0 | 0.0 |
| Caribbean | Belize | 0.1390 | WHO* | 53.4800 | estimate | 287699 | 0.4 | 153.9 |
| Caribbean | Bermuda | 0.0000 | N | 0.0000 | N | 83000 | 0.0 | 0.0 |
| Caribbean | British Virgin Islands | 0.0000 | N | 0.0000 | N | 22000 | 0.0 | 0.0 |
| Caribbean | Cayman Islands | 0.0000 | N | 0.0000 | N | 43000 | 0.0 | 0.0 |
| Caribbean | Cuba | 0.0000 | N | 0.0000 | N | 11267883 | 0.0 | 0.0 |
| Caribbean | Dominica | 0.0000 | N | 0.0000 | N | 67390 | 0.0 | 0.0 |
| Caribbean | Dominican Republic | 0.0000 | N | 0.0000 | N | 9759664 | 0.0 | 0.0 |
| Caribbean | French Guiana | 53.4800 | GL | 53.4800 | GL | 187000 | 100.0 | 100.0 |
| Caribbean | Grenada | 0.0000 | N | 0.0000 | N | 105672 | 0.0 | 0.0 |
| Caribbean | Guadeloupe | 0.0000 | N | 0.0000 | N | 446000 | 0.0 | 0.0 |
| Caribbean | Guyana | 0.5827 | GL | 53.4800 | estimate | 737906 | 4.3 | 394.6 |
| **Global Burden Region** | **Country** | **Mortality rate per 100,000 population (Lower)** | **Source** | **Mortality rate per 100,000 population (Higher)** | **Source** | **Current Population** | **Number of deaths (Low)** | **Number of deaths (High)** |
| Caribbean | Haiti | 0.0000 | N | 0.0000 | N | 9597849 | 0.0 | 0.0 |
| Caribbean | Jamaica | 0.0000 | N | 0.0000 | N | 2713779 | 0.0 | 0.0 |
| Caribbean | Martinique | 0.1390 | estimate | 53.4800 | estimate | 397000 | 0.6 | 212.3 |
| Caribbean | Montserrat | 0.0000 | N | 0.0000 | N | 4000 | 0.0 | 0.0 |
| Caribbean | Netherland Antilles | 0.0000 | N | 0.0000 | N | 224000 | 0.0 | 0.0 |
| Caribbean | Saint Kitts and Nevus | 0.0000 | N | 0.0000 | N | 50417 | 0.0 | 0.0 |
| Caribbean | Saint Lucia | 0.0000 | WHO* | 0.0000 | N | 164924 | 0.0 | 0.0 |
| Caribbean | Saint Vincent and Grenadine | 0.0000 | N | 0.0000 | N | 120402 | 0.0 | 0.0 |
| Caribbean | Suriname | 0.4367 | WHO* | 53.4800 | estimate | 457964 | 2.0 | 244.9 |
| Caribbean | Trinidad and Tobago | 0.0000 | WHO* | 0.0000 | WHO* | 1333272 | 0.0 | 0.0 |
| Caribbean | Turks and Caicos Island | 0.0000 | N | 0.0000 | N | 22000 | 0.0 | 0.0 |
| Europe, Central | Albania | 0.0000 | WHO* | 0.0000 | WHO* | 3190012 | 0.0 | 0.0 |
| Europe, Central | Bosnia & Herzegovina | 0.0000 | WHO* | 0.0000 | WHO* | 3934816 | 0.0 | 0.0 |
| Europe, Central | Bulgaria | 0.0000 | WHO* | 0.0000 | WHO* | 7638831 | 0.0 | 0.0 |
| Europe, Central | Croatia | 0.0220 | WHO* | 0.0220 | estimate | 4555398 | 1.0 | 1.0 |
| Europe, Central | Czech Republic | 0.0020 | WHO* | 0.0220 | estimate | 10186330 | 0.2 | 2.2 |
| Europe, Central | Hungary | 0.0020 | WHO* | 0.0220 | estimate | 10029683 | 0.2 | 2.2 |
| Europe, Central | Montenegro | 0.0020 | estimate | 0.0220 | estimate | 597983 | 0.0 | 0.1 |
| Europe, Central | Poland | 0.0068 | WHO* | 0.0220 | estimate | 38081971 | 2.6 | 8.4 |
| Europe, Central | Romania | 0.0000 | WHO* | 0.0000 | WHO* | 21437887 | 0.0 | 0.0 |
| Europe, Central | Serbia | 0.0101 | WHO* | 0.0220 | estimate | 9858424 | 1.0 | 2.2 |
| Europe, Central | Slovakia | 0.0111 | WHO* | 0.0220 | estimate | 5390035 | 0.6 | 1.2 |
| Europe, Central | Slovenia | 0.0000 | WHO* | 0.0000 | WHO* | 2001506 | 0.0 | 0.0 |
| Europe, Central | TFYR Macedonia | 0.0000 | WHO* | 0.0000 | WHO* | 2038464 | 0.0 | 0.0 |
| Europe, Eastern | Belarus | 0.0000 | WHO* | 0.0000 | WHO* | 9688795 | 0.0 | 0.0 |
| Europe, Eastern | Estonia | 0.0300 | WHO* | 0.0439 | estimate | 1335333 | 0.4 | 0.6 |
| Europe, Eastern | Latvia | 0.0439 | WHO* | 0.0439 | estimate | 2277040 | 1.0 | 1.0 |
| Europe, Eastern | Lithuania | 0.0059 | WHO* | 0.0439 | estimate | 3389937 | 0.2 | 1.5 |
| Europe, Eastern | Republic of Moldova | 0.0000 | WHO* | 0.0000 | WHO* | 3793604 | 0.0 | 0.0 |
| **Global Burden Region** | **Country** | **Mortality rate per 100,000 population (Lower)** | **Source** | **Mortality rate per 100,000 population (Higher)** | **Source** | **Current Population** | **Number of deaths (Low)** | **Number of deaths (High)** |
| Europe, Eastern | Russian Federation | 0.0246 | GL | 0.0439 | estimate | 142498532 | 35.0 | 62.6 |
| Europe, Eastern | Ukraine | 0.0000 | WHO* | 0.0000 | WHO* | 46205382 | 0.0 | 0.0 |
| Europe, Western | Andorra | 0.0005 | estimate | 0.0190 | estimate | 74601 | 0.0 | 0.0 |
| Europe, Western | Austria | 0.0000 | WHO* | 0.0000 | WHO* | 8360746 | 0.0 | 0.0 |
| Europe, Western | Belgium | 0.0000 | N | 0.0000 | N | 10457343 | 0.0 | 0.0 |
| Europe, Western | Channel Islands | 0.0000 | N | 0.0000 | N | 149000 | 0.0 | 0.0 |
| Europe, Western | Cyprus | 0.0000 | WHO* | 0.0000 | WHO* | 854671 | 0.0 | 0.0 |
| Europe, Western | Denmark | 0.0184 | WHO* | 0.0190 | WHO* | 5442098 | 1.0 | 1.0 |
| Europe, Western | Faro Islands | 0.0000 | N | 0.0000 | N | 47000 | 0.0 | 0.0 |
| Europe, Western | Finland | 0.0190 | WHO* | 0.0190 | WHO* | 5276892 | 1.0 | 1.0 |
| Europe, Western | France | 0.0016 | WHO* | 0.0190 | estimate | 61647375 | 1.0 | 11.7 |
| Europe, Western | Germany | 0.0005 | WHO* | 0.0190 | estimate | 82599471 | 0.4 | 15.7 |
| Europe, Western | Gibraltar | 0.0000 | N | 0.0000 | N | 27000 | 0.0 | 0.0 |
| Europe, Western | Greece | 0.0000 | WHO* | 0.0000 | WHO* | 11146918 | 0.0 | 0.0 |
| Europe, Western | Greenland | 0.0000 | N | 0.0000 | N | 57000 | 0.0 | 0.0 |
| Europe, Western | Holy See | 0.0000 | N | 0.0000 | N | 1000 | 0.0 | 0.0 |
| Europe, Western | Iceland | 0.0000 | N | 0.0000 | N | 301006 | 0.0 | 0.0 |
| Europe, Western | Ireland | 0.0000 | N | 0.0000 | N | 4300902 | 0.0 | 0.0 |
| Europe, Western | Isle of Man | 0.0000 | N | 0.0000 | N | 78000 | 0.0 | 0.0 |
| Europe, Western | Israel | 0.0029 | WHO* | 0.0190 | estimate | 6927677 | 0.2 | 1.3 |
| Europe, Western | Italy | 0.0000 | WHO* | 0.0000 | WHO* | 58876834 | 0.0 | 0.0 |
| Europe, Western | Liechtenstein | 0.0000 | N | 0.0000 | N | 34000 | 0.0 | 0.0 |
| Europe, Western | Luxembourg | 0.0000 | N | 0.0000 | N | 466560 | 0.0 | 0.0 |
| Europe, Western | Malta | 0.0000 | N | 0.0000 | N | 406582 | 0.0 | 0.0 |
| Europe, Western | Monaco | 0.0000 | N | 0.0000 | N | 32711 | 0.0 | 0.0 |
| Europe, Western | Netherlands | 0.0000 | WHO* | 0.0000 | WHO* | 16418824 | 0.0 | 0.0 |
| Europe, Western | Norway | 0.0000 | WHO* | 0.0000 | WHO* | 4698097 | 0.0 | 0.0 |
| Europe, Western | Portugal | 0.0000 | WHO* | 0.0000 | WHO* | 10623031 | 0.0 | 0.0 |
| Europe, Western | San Marino | 0.0000 | N | 0.0000 | N | 6000 | 0.0 | 0.0 |
| Europe, Western | San Pierre et Miquelon | 0.0000 | WHO* | 0.0000 | WHO* | 30926 | 0.0 | 0.0 |
| Europe, Western | Spain | 0.0000 | WHO* | 0.0000 | WHO* | 44279180 | 0.0 | 0.0 |
| Europe, Western | Sweden | 0.0022 | WHO* | 0.0190 | estimate | 9118955 | 0.2 | 1.7 |
| Europe, Western | Switzerland | 0.0067 | WHO* | 0.0190 | estimate | 7483973 | 0.5 | 1.4 |
| **Global Burden Region** | **Country** | **Mortality rate per 100,000 population (Lower)** | **Source** | **Mortality rate per 100,000 population (Higher)** | **Source** | **Current Population** | **Number of deaths (Low)** | **Number of deaths (High)** |
| Europe, Western | United Kingdom | 0.0007 | WHO* | 0.0190 | estimate | 60768946 | 0.4 | 11.5 |
| Latin America, Andean | Bolivia | 1.0499 | GL | 1.0499 | GL | 9524568 | 100.0 | 100.0 |
| Latin America, Andean | Ecuador | 0.3193 | GL | 1.0499 | estimate | 13341197 | 42.6 | 140.1 |
| Latin America, Andean | Peru | 0.3584 | GL | 1.0499 | estimate | 27902760 | 100.0 | 293.0 |
| Latin America, Central | Colombia | 0.1571 | WHO* | 0.7477 | estimate | 46155958 | 72.5 | 345.1 |
| Latin America, Central | Costa-Rica | 0.0851 | WHO* | 0.2000 | Rojas et al. 1997 | 4467625 | 3.8 | 8.9 |
| Latin America, Central | El Salvador | 0.0447 | estimate | 0.7477 | estimate | 6857328 | 3.1 | 51.3 |
| Latin America, Central | Guatemala | 0.0447 | estimate | 0.7477 | estimate | 13353911 | 6.0 | 99.9 |
| Latin America, Central | Honduras | 0.0447 | estimate | 0.7477 | estimate | 7106001 | 3.2 | 53.1 |
| Latin America, Central | Mexico | 0.0447 | WHO* | 0.7477 | estimate | 106534880 | 47.6 | 796.6 |
| Latin America, Central | Nicaragua | 0.0447 | estimate | 0.7477 | estimate | 5603190 | 2.5 | 41.9 |
| Latin America, Central | Panama | 0.5204 | WHO* | 0.7477 | GL | 3343374 | 17.4 | 25.0 |
| Latin America, Central | Venezuela | 0.1338 | WHO* | 0.1900 | De Sousa et al. 2005 | 27656832 | 37.0 | 38.8 |
| Latin America, Southern | Argentina | 0.0095 | WHO* | 0.0095 | WHO* | 39531115 | 3.8 | 3.8 |
| Latin America, Southern | Chile | 0.0012 | WHO* | 0.0095 | estimate | 16634760 | 0.2 | 1.6 |
| Latin America, Southern | Falkland Islands | 0.0000 | N | 0.0000 | N | 3000 | 0.0 | 0.0 |
| Latin America, Southern | Uruguay | 0.0000 | WHO* | 0.0000 | WHO* | 3339700 | 0.0 | 0.0 |
| Latin America, Tropical | Brazil | 0.0488 | WHO* | 0.1512 | GL | 191790929 | 93.5 | 290.0 |
| Latin America, Tropical | Paraguay | 0.1142 | GL | 0.1512 | estimate | 6127077 | 7.0 | 9.3 |
| North Africa/ Middle East | Algeria | 0.0084 | estimate | 0.0185 | estimate | 33857915 | 2.9 | 6.3 |
| North Africa/ Middle East | Bahrain | 0.0000 | WHO* | 0.0000 | WHO* | 752648 | 0.0 | 0.0 |
| North Africa/ Middle East | Egypt | 0.0185 | GL | 0.0185 | GL | 75497913 | 14.0 | 14.0 |
| North Africa/ Middle East | Iran | 0.0084 | estimate | 0.0185 | estimate | 71208384 | 6.0 | 13.2 |
| North Africa/ Middle East | Iraq | 0.0084 | estimate | 0.0185 | estimate | 28993374 | 2.4 | 5.4 |
| North Africa/ Middle East | Jordan | 0.0084 | GL | 0.0185 | estimate | 5924245 | 0.5 | 1.1 |
| North Africa/ Middle East | Kuwait | 0.0000 | WHO* | 0.0000 | WHO* | 2851144 | 0.0 | 0.0 |
| North Africa/ Middle East | Lebanon | 0.0084 | estimate | 0.0185 | estimate | 4099115 | 0.3 | 0.8 |
| North Africa/ Middle East | Libyan Arab Jamahiriya | 0.0084 | estimate | 0.0185 | estimate | 6160483 | 0.5 | 1.1 |
| North Africa/ Middle East | Morocco | 0.0084 | estimate | 0.0185 | estimate | 31224137 | 2.6 | 5.8 |
| North Africa/ Middle East | Occupied Palestinian Territory | 0.0084 | estimate | 0.0185 | estimate | 3762000 | 0.3 | 0.7 |
| **Global Burden Region** | **Country** | **Mortality rate per 100,000 population (Lower)** | **Source** | **Mortality rate per 100,000 population (Higher)** | **Source** | **Current Population** | **Number of deaths (Low)** | **Number of deaths (High)** |
| North Africa/ Middle East | Oman | 0.0084 | estimate | 0.0185 | estimate | 2595133 | 0.2 | 0.5 |
| North Africa/ Middle East | Qatar | 0.0000 | WHO* | 0.0000 | WHO* | 840635 | 0.0 | 0.0 |
| North Africa/ Middle East | Saudi Arabia | 0.0084 | estimate | 0.0185 | estimate | 24734533 | 2.1 | 4.6 |
| North Africa/ Middle East | Syrian Arab Republic | 0.0084 | estimate | 0.0185 | estimate | 19928516 | 1.7 | 3.7 |
| North Africa/ Middle East | Tunisia | 0.0084 | estimate | 0.0185 | estimate | 10327285 | 0.9 | 1.9 |
| North Africa/ Middle East | Turkey | 0.0084 | estimate | 0.0185 | estimate | 74876695 | 6.3 | 13.9 |
| North Africa/ Middle East | United Arab Emirates | 0.0084 | estimate | 0.0185 | estimate | 4380439 | 0.4 | 0.8 |
| North Africa/ Middle East | Western Sahara | 0.0084 | estimate | 0.0185 | estimate | 324000 | 0.0 | 0.1 |
| North Africa/ Middle East | Yemen | 0.0084 | estimate | 0.0185 | estimate | 22389169 | 1.9 | 4.2 |
| North America, High income | Canada | 0.0000 | WHO* | 0.0000 | WHO* | 32876047 | 0.0 | 0.0 |
| North America, High income | USA | 0.0016 | WHO* | 0.0230 | Forrester & Stanley 2004 | 305826246 | 4.8 | 7.0 |
| Oceania | American Samoa | 0.0000 | N | 0.0000 | N | 65000 | 0.0 | 0.0 |
| Oceania | Cook Islands | 0.0000 | N | 0.0000 | N | 13325 | 0.0 | 0.0 |
| Oceania | Fiji | 0.0000 | N | 0.0000 | N | 838698 | 0.0 | 0.0 |
| Oceania | French Polynesia | 0.0000 | N | 0.0000 | N | 252000 | 0.0 | 0.0 |
| Oceania | Guam | 0.0000 | N | 0.0000 | N | 168000 | 0.0 | 0.0 |
| Oceania | Kiribati | 0.0000 | N | 0.0000 | N | 95067 | 0.0 | 0.0 |
| Oceania | Marshall Islands | 0.0000 | N | 0.0000 | N | 59286 | 0.0 | 0.0 |
| Oceania | Micronesia (Federated States of) | 3.1590 | estimate | 7.9000 | estimate | 111117 | 3.5 | 7.2 |
| Oceania | Nauru | 0.0000 | N | 0.0000 | N | 10152 | 0.0 | 0.0 |
| Oceania | New Caledonia | 0.0000 | N | 0.0000 | N | 237000 | 0.0 | 0.0 |
| Oceania | Niue | 3.1590 | estimate | 7.9000 | estimate | 1565 | 0.0 | 0.1 |
| Oceania | Northern Mariana Islands | 0.0000 | N | 0.0000 | N | 86000 | 0.0 | 0.0 |
| Oceania | Palau | 3.1590 | estimate | 7.9000 | estimate | 20314 | 0.6 | 1.0 |
| Oceania | Papua NG | 3.1590 | GL | 7.9000 | Lalloo et al. 1999 | 6331010 | 200.0 | 455.7 |
| Oceania | Pitcairn Islands | 0.0000 | N | 0.0000 | N | 0 | 0.0 | 0.0 |
| Oceania | Samoa | 0.0000 | N | 0.0000 | N | 187023 | 0.0 | 0.0 |
| **Global Burden Region** | **Country** | **Mortality rate per 100,000 population (Lower)** | **Source** | **Mortality rate per 100,000 population (Higher)** | **Source** | **Current Population** | **Number of deaths (Low)** | **Number of deaths (High)** |
| Oceania | Solomon Islands | 3.1590 | estimate | 7.9000 | estimate | 495662 | 15.7 | 36.5 |
| Oceania | Tokelau | 0.0000 | N | 0.0000 | N | 2000 | 0.0 | 0.0 |
| Oceania | Tonga | 0.0000 | WHO* | 0.0000 | N | 100336 | 0.0 | 0.0 |
| Oceania | Tuvalu | 0.0000 | N | 0.0000 | N | 10530 | 0.0 | 0.0 |
| Oceania | Vanuatu | 3.1590 | estimate | 7.9000 | estimate | 226180 | 7.1 | 15.6 |
| Oceania | Wallis and Futuna Islands | 0.0000 | N | 0.0000 | N | 15000 | 0.0 | 0.0 |
| Sub Saharan Africa, Central | Angola | 0.1149 | estimate | 5.5339 | estimate | 17024084 | 19.6 | 542.5 |
| Sub Saharan Africa, Central | Central African Republic | 0.1149 | estimate | 5.5339 | estimate | 4342735 | 5.0 | 130.1 |
| Sub Saharan Africa, Central | Congo | 0.1149 | Chippaux 2005§ | 3.9105 | Carme et al. 1986§ | 3768086 | 4.3 | 75.2 |
| Sub Saharan Africa, Central | DR Congo | 0.3581 | Odio et al. 2005§ | 5.5339 | Bokata 2005§ | 62635723 | 224.3 | 2305.9 |
| Sub Saharan Africa, Central | Equatorial Guinea | 0.1149 | estimate | 5.5339 | estimate | 507499 | 0.6 | 15.4 |
| Sub Saharan Africa, Central | Gabon | 0.1729 | Chippaux 2005§ | 5.5339 | estimate | 1330574 | 2.3 | 13.6 |
| Sub Saharan Africa, East | Burundi | 0.4500 | estimate | 4.4659 | estimate | 8508232 | 38.3 | 322.7 |
| Sub Saharan Africa, East | Comoros | 0.0000 | N | 0.0000 | N | 839187 | 0.0 | 0.0 |
| Sub Saharan Africa, East | Djibouti | 0.4500 | estimate | 4.4659 | estimate | 833021 | 3.7 | 8.4 |
| Sub Saharan Africa, East | Eritrea | 0.4500 | estimate | 4.4659 | estimate | 4850763 | 21.8 | 173.7 |
| Sub Saharan Africa, East | Ethiopia | 0.4500 | estimate | 4.4659 | estimate | 83099190 | 373.9 | 3002 |
| Sub Saharan Africa, East | Kenya | 0.4500 | Coombs et al. 1997 | 4.4659 | Snow et al. 1994§ | 37537716 | 168.9 | 980.8 |
| Sub Saharan Africa, East | Madagascar | 0.0000 | N | 0.0000 | N | 19683358 | 0.0 | .0 |
| Sub Saharan Africa, East | Malawi | 0.4500 | estimate | 4.4659 | estimate | 13925070 | 62.7 | 503.0 |
| Sub Saharan Africa, East | Mozambique | 0.4500 | estimate | 4.4659 | estimate | 21396916 | 96.3 | 602.5 |
| Sub Saharan Africa, East | Rwanda | 0.4500 | estimate | 4.4659 | estimate | 9724577 | 43.8 | 328.5 |
| Sub Saharan Africa, East | Somalia | 0.4500 | estimate | 4.4659 | estimate | 8698534 | 39.1 | 332.4 |
| Sub Saharan Africa, East | Sudan | 0.4500 | estimate | 4.4659 | estimate | 38560488 | 173.5 | 1044.4 |
| Sub Saharan Africa, East | Uganda | 0.4500 | estimate | 4.4659 | estimate | 30883805 | 139.0 | 1175.5 |
| Sub Saharan Africa, East | United Republic of Tanzania | 0.4500 | estimate | 4.4659 | estimate | 40453513 | 182.0 | 1194.4 |
| Sub Saharan Africa, East | Zambia | 0.4500 | estimate | 4.4659 | estimate | 11921999 | 53.6 | 346.2 |
| **Global Burden Region** | **Country** | **Mortality rate per 100,000 population (Lower)** | **Source** | **Mortality rate per 100,000 population (Higher)** | **Source** | **Current Population** | **Number of deaths (Low)** | **Number of deaths (High)** |
| Sub Saharan Africa, South | Botswana | 0.1423 | estimate | 0.5300 | estimate | 1881504 | 2.7 | 10.0 |
| Sub Saharan Africa, South | Lesotho | 0.1423 | estimate | 0.5300 | estimate | 2007833 | 2.9 | 10.6 |
| Sub Saharan Africa, South | Namibia | 0.1423 | estimate | 0.5300 | estimate | 2074146 | 3.0 | 11.0 |
| Sub Saharan Africa, South | South Africa | 0.5300 | McNally & Reitz 1987 | 0.5300 | McNally & Reitz 1987 | 48576763 | 257.5 | 257.5 |
| Sub Saharan Africa, South | Swaziland | 0.1423 | estimate | 0.5300 | estimate | 1141427 | 1.6 | 6.0 |
| Sub Saharan Africa, South | Zimbabwe | 0.1423 | GL | 0.5300 | estimate | 13349434 | 19.0 | 70.8 |
| Sub Saharan Africa, West | Benin | 7.1960 | Chippaux 2005 | 9.9969 | Chippaux 2005 | 9032787 | 650.0 | 764.1 |
| Sub Saharan Africa, West | Burkina Faso | 1.3528 | Chippaux 2005 | 3.3813 | Chippaux 2005 | 14784291 | 200.0 | 441.9 |
| Sub Saharan Africa, West | Cameroon | 0.1822 | Chippaux 2005 | 3.0389 | Chippaux 2005 | 18549176 | 33.8 | 265.9 |
| Sub Saharan Africa, West | Cape Verde | 0.0000 | N | 0.0000 | N | 530437 | 0.0 | 0.0 |
| Sub Saharan Africa, West | Chad | 0.0324 | estimate | 14.0000 | estimate | 10780571 | 3.5 | 1008.1 |
| Sub Saharan Africa, West | Cote d’Ivoire | 1.0383 | Chippaux 2002 | 1.5575 | Chippaux 2002 | 19261821 | 200.0 | 300.0 |
| Sub Saharan Africa, West | Gambia | 0.0324 | estimate | 14.0000 | estimate | 1708681 | 0.6 | 163.9 |
| Sub Saharan Africa, West | Ghana | 1.8613 | GL§ | 14.0000 | estimate | 23478394 | 7.6 | 1713.3 |
| Sub Saharan Africa, West | Guinea-Bissau | 0.0324 | estimate | 14.0000 | estimate | 1695043 | 0.5 | 151.8 |
| Sub Saharan Africa, West | Guinea | 0.2999 | Balde et al 2002§ | 4.9999 | Balde et al 2002§ | 9370113 | 28.1 | 468.5 |
| Sub Saharan Africa, West | Liberia | 0.0324 | estimate | 14.0000 | estimate | 3750261 | 1.2 | 286.9 |
| Sub Saharan Africa, West | Mali | 0.0324 | Chippaux 2005 | 14.0000 | estimate | 12336799 | 4.0 | 1365.5 |
| Sub Saharan Africa, West | Mauritania | 0.0324 | estimate | 14.0000 | estimate | 3123813 | 1.0 | 162.3 |
| Sub Saharan Africa, West | Niger | 0.1877 | Chippaux & Kambewasso 2002 | 0.6580 | Chippaux & Kambewasso 2002 | 14225521 | 26.7 | 76.5 |
| Sub Saharan Africa, West | Nigeria | 0.0324 | estimate | 14.0000 | estimate | 148092542 | 48.0 | 9900.9 |
| Sub Saharan Africa, West | Saint Helena | 0.0324 | estimate | 14.0000 | estimate | 5000 | 0.0 | 0.4 |
| Sub Saharan Africa, West | Sao Tome and Principe | 0.0324 | estimate | 14.0000 | estimate | 157638 | 0.1 | 15.2 |
| Sub Saharan Africa, West | Senegal | 1.5000 | Chippaux & Diallo 2002 | 14.0000 | Trape et al. 2001 | 12378532 | 185.7 | 867.0 |
| **Global Burden Region** | **Country** | **Mortality rate per 100,000 population (Lower)** | **Source** | **Mortality rate per 100,000 population (Higher)** | **Source** | **Current Population** | **Number of deaths (Low)** | **Number of deaths (High)** |
| Sub Saharan Africa, West | Sierra Leone | 0.0324 | estimate | 14.0000 | estimate | 5865872 | 1.9 | 470.1 |
| Sub Saharan Africa, West | Togo | 3.0000 | Chippaux 2005 | 4.0000 | Chippaux 2005 | 6585147 | 197.6 | 232.0 |

WHO* - WHO Mortality Database	GL- Grey literature	§ - Extrapolated using CFR	N- Countries without snakebite
